# The preparation of [1,2,4]triazolo[1,5-*a*]pyrimidines catalyzed by Schiff base zinc(ii) complex supported on magnetite nanoparticles under mild conditions†

**DOI:** 10.1039/d4ra02339k

**Published:** 2024-06-14

**Authors:** Ahmad Reza Moosavi-Zare, Raha Najafi, Hamid Goudarziafshar

**Affiliations:** a Department of Chemical Engineering, Hamedan University of Technology Hamedan 65155 Iran moosavizare@yahoo.com; b Chemistry Department, College of Sciences, Shiraz University Shiraz 71946-84795 Iran

## Abstract

Nano-[CuFe_2_O_4_@SiO_2_/propyl-1-(*O*-vanillinaldimine)][ZnCl_2_] was prepared by placing a Schiff base zinc(ii) complex on a magnetite core and fully characterized by various analyses such as FT-IR, FE-SEM, EDAX, SEM-coupled EDX, TGA, VSM and TEM. The complexes supported on silica-coated magnetite copper ferrite nanoparticles were used as a reusable catalyst for the synthesis of 5-methyl-*N*,7-diphenyl-4,7-dihydro-[1,2,4]triazolo[1,5-*a*] pyrimidine-6-carboxamides resulting in 40% to 96% yield in the reactions of various aldehydes, acetoacetanilide, and 3-amino-1,2,4-triazole at 60 °C under solvent-free conditions. The zinc complex can change its structure from tetrahedral to square planar and catalyze the reaction. Some products containing the benzyloxy moiety are new and have been reported for the first time.

## Abstract 2

Nano-[CuFe_2_O_4_@SiO_2_/propyl-1-(*O*-vanillinaldimine)][ZnCl_2_] was prepared by the placement of Schiff base zinc(ii) complex on the magnetite core and used as a reusable catalyst for the synthesis of 5-methyl-*N*,7-diphenyl-4,7-dihydro-[1,2,4]triazolo[1,5-*a*] pyrimidine-6-carboxamide derivatives.

## Graphical abstract

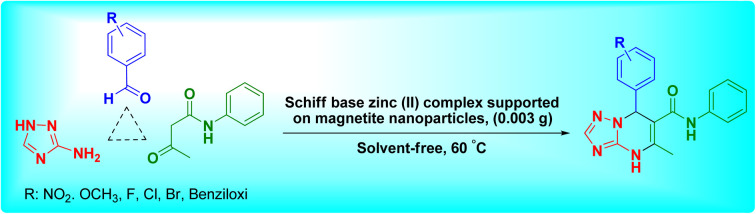

## 1 Introduction

Recently, the preparation and application of nano magnetic materials in various domains of sciences has attracted the attention of scientists. Nano magnetic materials have been used as effective catalysts to promote organic transformations. When using catalysts supported on the magnetite materials, the recovery of the catalyst can be done easily as the catalyst can be simply separated from the reaction mixture.^1–8^

Heterogeneous catalysts are an important group of catalysts with characteristics such as high stability, high active surface, existence in phases different from those of starting materials and products, ease of isolation and recovery, and use on a large scale.^9–12^ One of the categories of the catalysts with all these features is the magnetic catalyst, or catalyst supported on a magnetic surface. Such catalysts are also easily attracted by an external magnet from the reaction mixture owing to their magnetic properties.^6,7,13,14^ One of these catalysts that has the potential to be bonded to a magnetic surface is Schiff base complexes. One of the most important features of catalysts supported on magnetic surfaces is the ease of separation from the reaction mixture and potential for reuse in other reactions, which reduces material consumption and enhances atomic economy.^6,7^ Schiff base complexes comprise an organic part and a metal, whereby the organic part is a ligand, which is formed from the combination of a primary amine and a carbonyl compound. Some Schiff base ligands have medicinal properties such as antifungal,^15,16^ antibacterial,^17^ antiviral^18^ and antioxidant properties.^18^ Such complexes have also been used as sensors to absorb some metal ions and as catalysts in many chemical reactions.^18–23^

Today, multi-component reactions are considered as an efficient method in organic synthesis and the production of biological compounds and essential drugs. This protocol has some important advantages such as reduction of energy and time consumption, high efficiency, easy purification of the products and reduction of waste material production without the production of side products. There is also no need to separate the produced intermediates.^24–31^

Phenyl-[1,2,4]triazolo[1,5-*a*]pyrimidine-6-carboxamide compounds have some significant activities and are widely used in medicinal chemistry.^32^ Triazolopyrimidine derivatives are known to be blood pressure regulators,^33^ antibacterial agents,^34^ anticancer agents,^35–37^ antidiabetics agents,^38^ antiproliferative agents,^39^ have anti-tumor activity,^40^ protein kinase inhibitors,^41^ antifungal agents,^42^ and macrophage activators.^43,44^ Phenyl-[1,2,4]triazolo[1,5-*a*]pyrimidine-6-carboxamide has been previously produced through the multi-component synthesis of acetoacetanylide, 3-amino[1,2,4]triazole and aldehydes using various catalysts such as *p*-toluenesulfonic acid,^45^ HCl,^46^ maltose,^47^ and triethylaminium-*N*-sulfonic acid tetrachloroaluminate.^48^ Considering the importance of this category of triazolopyrimidine compounds in medicinal chemistry, new preparation methods are required for them.

**Scheme 1:**
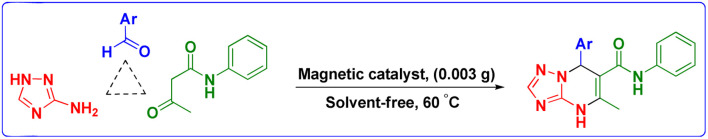
The preparation of [1,2,4]triazolo[1,5-*a*]pyrimidines.

Keeping this need in mind, we prepared a new Schiff base zinc(ii) complex supported on magnetite nanoparticles and successfully used it as a heterogeneous catalyst for the preparation of phenyl-[1,2,4]triazolo[1,5-*a*]pyrimidine-6-carboxamide at 60 °C under solvent-free conditions (Scheme 1).

## 2 Experimental

### 2.1. Materials

All materials were purchased from Merck and used without further purification; their purity was, however, checked by thin-layer chromatography (TLC).

### 2.2. Instrumental

The melting point of products were measured by electrothermal IA 9100 device. The FT-IR spectra of compounds were recorded by a Thermo device (model Avatar) spectrometer. ^1^H NMR and ^13^C NMR spectra were recorded on Bruker DRX-250 Avance with DMSO as a solvent.

### 2.3. Catalyst synthesis

#### 2.3.1 General method for the synthesis of CuFe_2_O_4_

To a mixture of FeCl_3_·6H_2_O (1.0 mmol) and CuCl_2_·2H_2_O (0.5 mmol), 5 ml of NaOH (0.2 M) and 9 ml of distilled water were added, and the reaction mixture was stirred under an atmosphere of nitrogen for 5 h at 90 °C. After this time, the prepared particles were filtered, dried, and calcinated in a thermal oven at a 700 °C for 5 h.

#### 2.3.2 General method for the synthesis of CuFe_2_O_4_@SiO_2_ core–shell

CuFe_2_O_4_ nanoparticles (2 g) were dispersed in a mixture of ethanol and water (200 : 10 v/v) using an ultrasonic bath for 10 minutes. Then, 6.64 ml of ammonia solution (25%) and tetramethyl orthosilicate (2 ml) were added dropwise to the mixture and stirred for 24 hours at room temperature. Finally, the obtained CuFe_2_O_4_@SiO_2_ nanoparticles were heated at 60 °C in vacuum oven for 3 hours to remove the solvent.

#### 2.3.3 Synthesis of (OEt)_3_Si/propyl-1-(*O*-vanillinaldimine)

(3-Aminopropyl)triethoxysilane (1.0 mmol) and *ortho*-vanillin (1.0 mmol) were added into a round-bottomed flask connected to a reflux condenser and stirred at room temperature for 24 hours, under solvent-free conditions. Finally, the obtained product was dried at room temperature as a Schiff base ligand. The structure of the Schiff base ligand was confirmed by FT-IR and ^1^H NMR.

#### 2.3.4 The metallization of (OEt)_3_Si/propyl-1-(*O*-vanillinaldimine)

To a round-bottomed flask containing the prepared ligand (2.0 mmol) and zinc chloride (1.0 mmol), a few drops of ethanol as a solvent were added and stirred at 70 °C for 24 hours to yield the Schiff base complex.

#### 2.3.5 Synthesis of nano [CuFe_2_O_4_@SiO_2_/propyl-1-(*O*-vanillinaldimine*)*][ZnCl_2_]

CuFe_2_O_4_@SiO_2_ nanoparticles (2 g) were dispersed in toluene (15 ml) for 20 minutes. Then, the prepared Schiff base complex (1.0 mmol) was added to the mixture and stirred for 24 hours under reflux conditions at 110 °C. Finally, the obtained nanomagnetic Schiff base complex was collected by an external magnet and washed with ethanol (15 ml) (three times) and dried at 60 °C.

### 2.4. Catalytic tests

#### 2.4.1 General method for the synthesis of [1,2,4]triazolo[1,5-*a*]pyrimidine-6-carboxamide

A mixture of 3-amino-1,2,4-triazole (1.0 mmol), aromatic aldehyde (1.0 mmol), and acetoacetanilide (1.0 mmol) in the presence of CuFe_2_O_4_@SiO_2_/propyl-1-(*O*-) vanillinaldimine [ZnCl_2_] as a nano magnetite catalyst (0.003 g) was stirred at 60 °C for 25 minutes. The reaction proceedings were monitored by TLC. After reaction completion, EtOH was added to the reaction mixture and stirred for an appropriate time period. The precipitate was then filtered and recrystallized from EtOH (95%) to obtain the pure product.

## 3 Results and discussion

### 3.1. Synthesis and characterization of catalyst

**Scheme 2:**
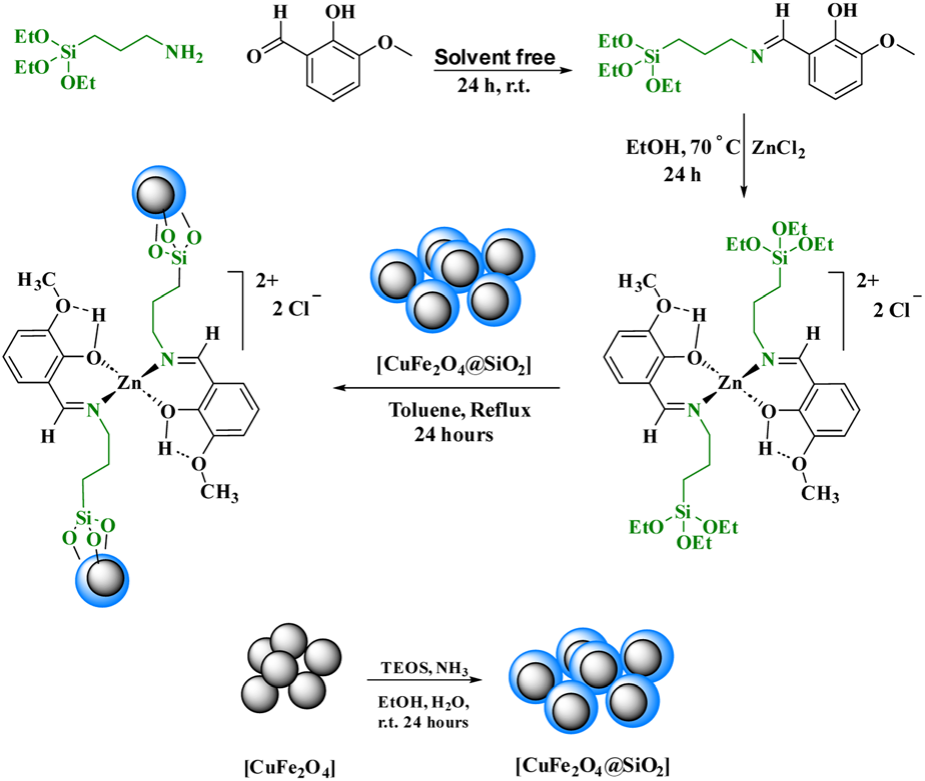
The preparation of nano-[CuFe_2_O_4_@SiO_2_/propyl-1-(*O*-vanillinaldimine)][ZnCl_2_].

In this research, a Schiff base complex of zinc(ii) was synthesized and supported on magnetite nanoparticles for use as a reusable catalyst in organic reactions. To create the catalyst, a Schiff base ligand was first synthesized by the reaction of 3-aminopropyl triethoxysilane with ortho-vanillin. Metalation of the presented ligand was then performed by adding ZnCl_2_ in dry toluene to give (OEt)_3_Si/propyl-1-(*O*-vanillinaldimine) as a Schiff base complex. The prepared Schiff base complex was the adsorbed on CuFe_2_O_4_@SiO_2_ nanoparticles to obtain nano [CuFe_2_O_4_@SiO_2_/propyl-1-(*O*-vanillinaldimine)][ZnCl_2_] as a nano magnetite Schiff base complex (Scheme 2).

**Fig. 1:**
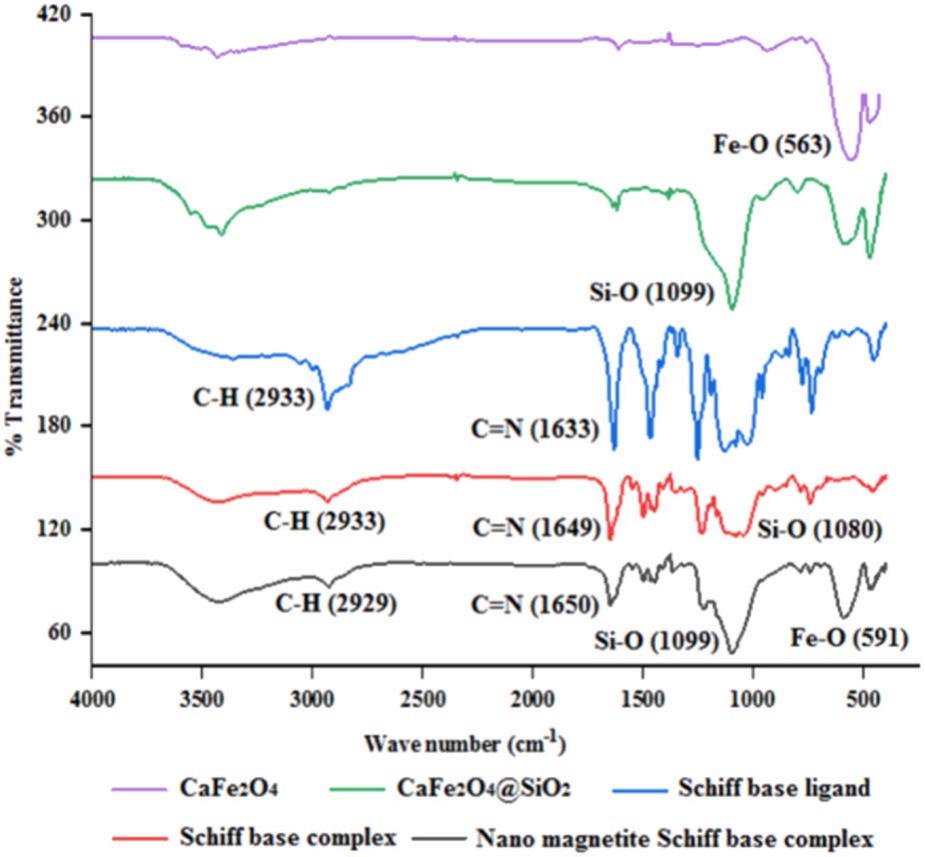
The FT-IR spectrum of nano magnetic catalyst in comparison with other species in the catalytic structure.

To show the specific bonds in the structure of the supported catalyst, the FT-IR spectrum of [CuFe_2_O_4_@SiO_2_/propyl-1-(*O*-vanillinaldimine)][ZnCl_2_] was recorded and the important peaks were studied (Fig. 1). As shown in Fig. 1, the peaks at about 591 and 1099 cm^−1^ are related to Fe–O and Si–O bond vibrations, respectively. The peak at about 2929 cm^−1^ corresponds to the C–H bond vibration. Moreover, the peak at about 1650 cm^−1^ confirmed the presence of a C=N bond in the Schiff base ligand in [CuFe_2_O_4_@SiO_2_/propyl-1-(*O*-vanillinaldimine)][ZnCl_2_], which is coordinated with zinc chloride (Fig. 1). In Fig. 1, the FT-IR spectrum of the catalyst was compared with other species in the structure of the catalyst to show the changes that had occurred in its structure.

**Fig. 2:**
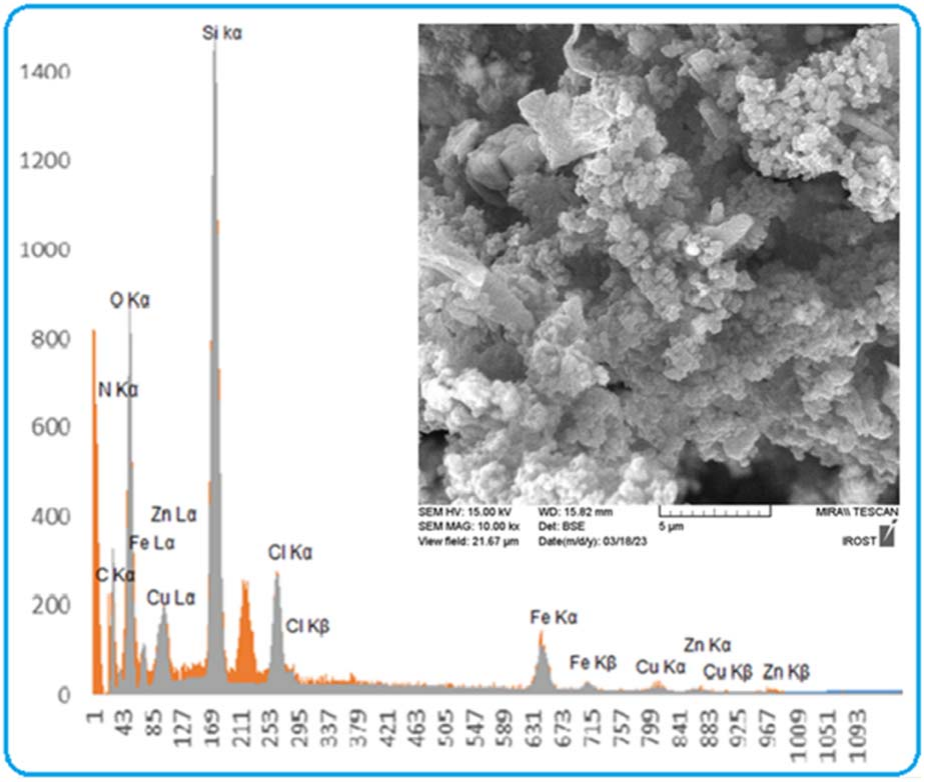
The EDX analysis of nano magnetite Schiff base complex.

Energy-dispersive X-ray spectroscopy (EDX) analysis was used to determine the elements present in the structure of [CuFe_2_O_4_@SiO_2_/propyl-1-(*O*-vanillinaldimine)][ZnCl_2_]. From the results of this analysis, it was determined that the structure contains various elements, including copper, iron, oxygen, nitrogen, carbon, silicon, zinc and chlorine (Fig. 2).

**Fig. 3:**
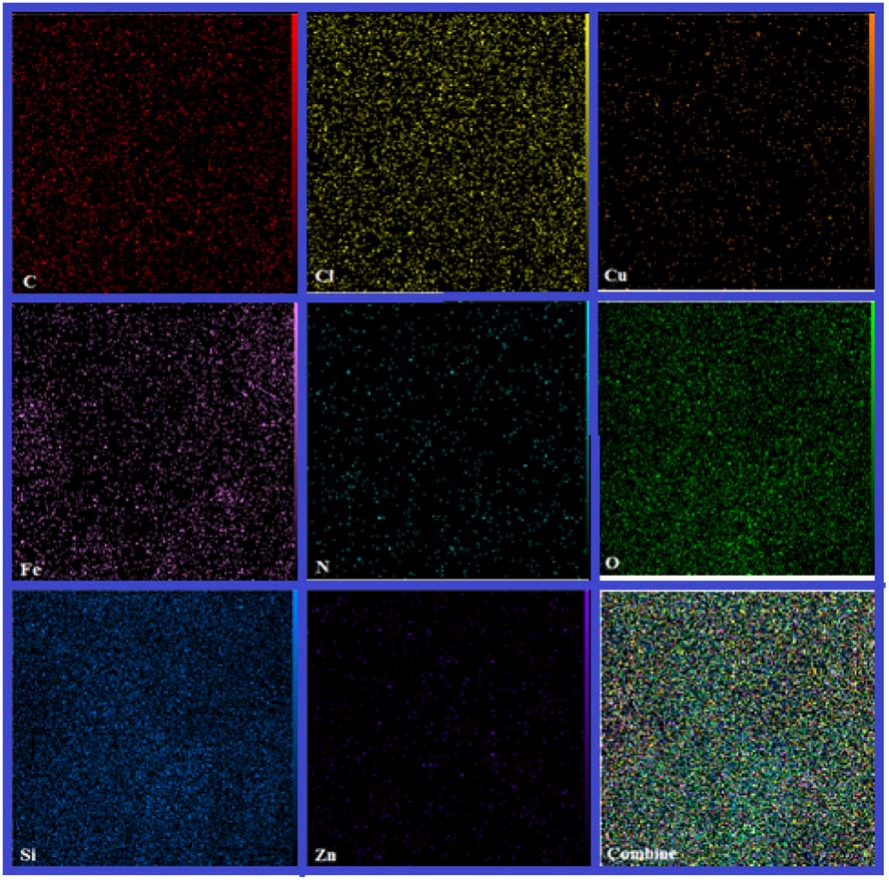
SEM-coupled EDX (SEM mapping) of the nano magnetite Schiff base complex.

SEM-coupled EDX (SEM mapping) was among other analyses conducted to determine the elements present and how they are distributed in the structure of the catalyst. The relevant images are shown in Fig. 3.

**Fig. 4:**
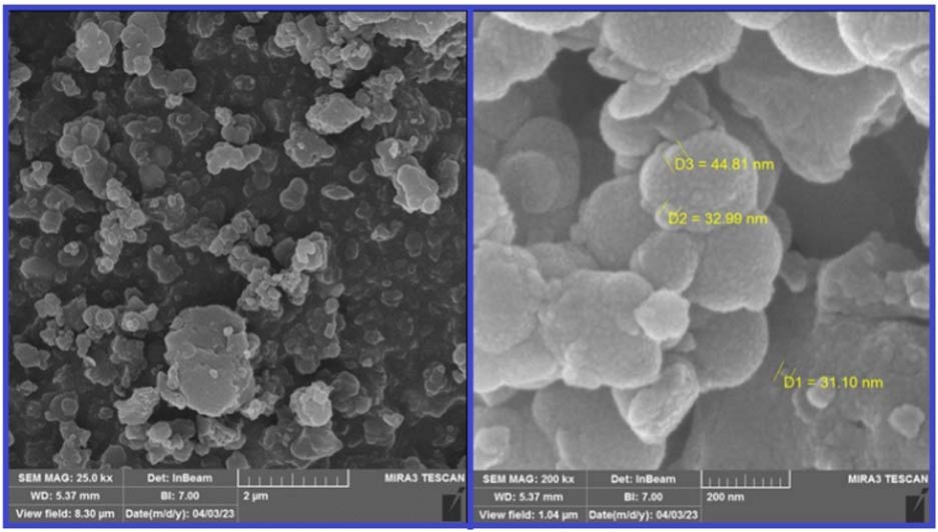
The FE-SEM analysis of nano magnetite Schiff base complex.

Field-emission scanning electron microscopy (FE-SEM) analysis was used to check the morphology and size of the particles on the surface of the catalyst. From this investigation, it was found that the particles were formed in sizes <100 nm (Fig. 4).

**Fig. 5:**
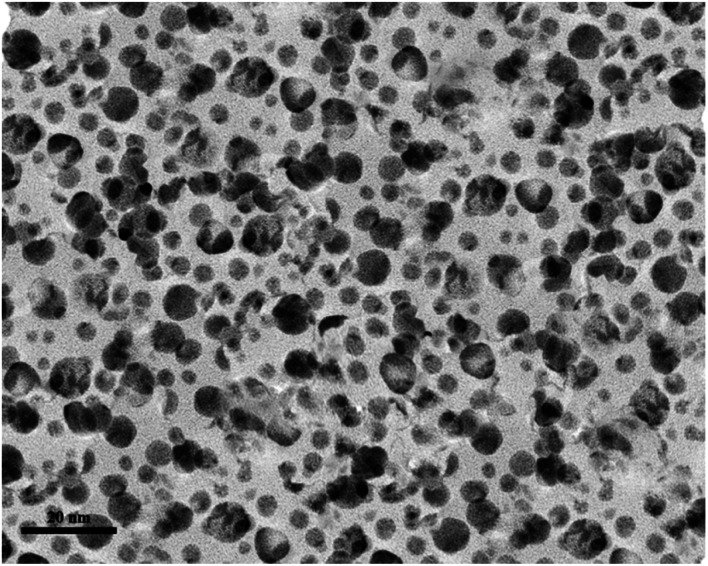
TEM image of nano magnetite Schiff base complex.

To find the particle size distribution of the catalyst, transmission electron microscopy (TEM) of [CuFe_2_O_4_@SiO_2_/propyl-1-(*O*-vanillinaldimine)][ZnCl_2_] was used, and showed that the catalyst particles were nano size (Fig. 5).

**Fig. 6:**
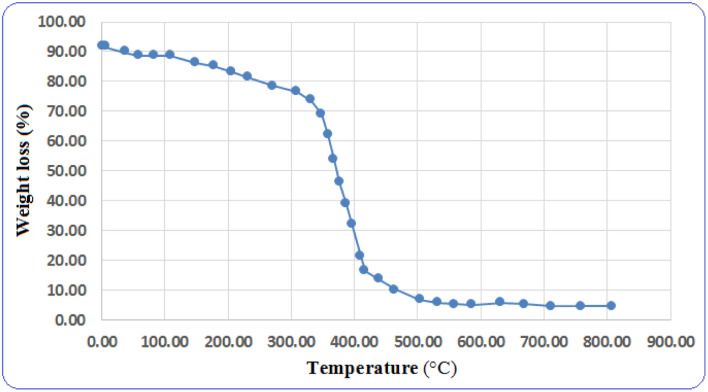
Thermal gravimetric analysis (TGA) of the nano magnetite Schiff base complex.

To determine the thermal stability of the catalyst and its usability in various reactions, thermal gravimetric analysis (TGA) was performed on the catalyst. From the results obtained from this analysis, the designed catalyst can be used in temperatures of up to 300 °C (Fig. 6). Based on the results obtained from the TGA analysis, this catalyst shows acceptable thermal stability in chemical reactions.

**Fig. 7:**
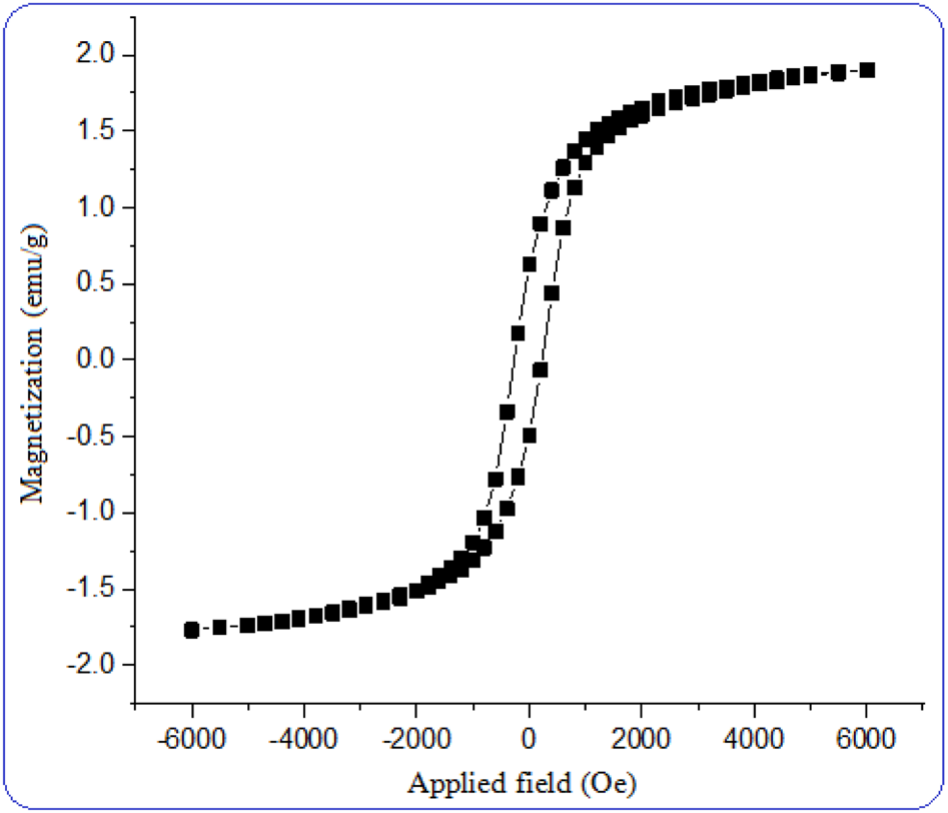
Vibrating sample magnetometer (VSM) analysis of nano magnetite Schiff base complex.

The magnetic behavior of the nano magnetite catalyst was measured using a vibrating sample magnetometer (VSM) at room temperature. The saturation magnetization for [CuFe_2_O_4_@SiO_2_/propyl-1-(*O*-vanillinaldimine)][ZnCl_2_] was found to be 2 emu g^−1^. The related hysteresis loops of the nano magnetite catalyst are displayed in Fig. 7. The magnetic ability of this catalyst enables it to be separated from the reaction mixture and reused.

### 3.2. Reaction of 4-nitrobenzaldehyde with acetoacetanilide and 3-amino-1,2,4-triazole

To verify the catalytic ability of [CuFe_2_O_4_@SiO_2_/propyl-1-(*O*-vanillinaldimine)][ZnCl_2_] in chemical reactions, this catalyst was used in the synthesis of [1,2,4]triazolo[1,5-*a*]pyrimidines. At first, to find the best reaction conditions, the reaction between 4-nitrobenzaldehyde with acetoacetanilide and 3-amino-1,2,4-triazole was chosen as a model reaction and the effects of the amount of catalyst, solvent and temperature were studied in this reaction (Table 1). As indicated in Table 1, the best result was achieved in the presence of 0.003 g of the catalyst at 60 °C under solvent-free conditions. Various solvents such as CHCl_3_, ethanol, ethyl acetate, and *n*-hexane were used in this reaction in comparison with the solvent-free condition, all of which did not have acceptable results.

**Table 1:** The effect of temperatures, solvents and various amount of catalyst on the reaction of 4-nitrobenzaldehyde with acetoacetanilide and 3-amino-1,2,4-triazole

Entry	Solvent	Catalyst (g)	Temp. (°C)	Time (min)	Yield^a^ (%)
1	Solvent free	0.003	60	25	93
2	Solvent free	—	60	25	Trace
3	Solvent free	0.003	r.t.	25	Trace
4	Solvent free	0.003	80	25	82
5	Solvent free	0.001	60	25	60
6	Solvent free	0.005	60	25	90
7	CHCl_3_	0.003	60	25	18
8	Ethanol	0.003	60	25	60
9	Ethyl acetate	0.003	60	25	31
10	*n*-Hexane	0.003	60	25	16

a Isolated yield.

### 3.3. Synthesis of [1,2,4]triazolo[1,5-*a*]pyrimidines

**Scheme 3:**
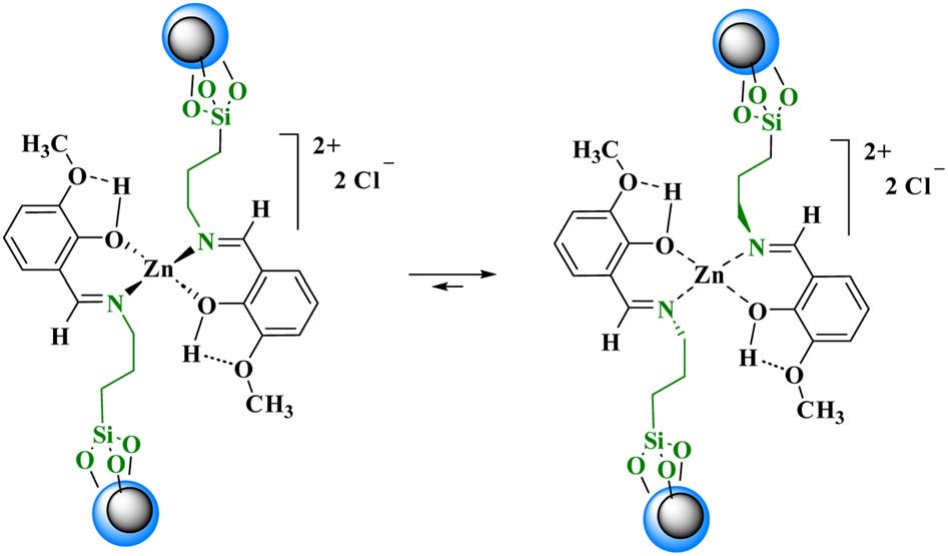
The transformation from a tetrahedral to a square planar for zinc complex.

After the optimization of the reaction conditions, various aryl aldehydes containing electron-withdrawing groups, electron-donating groups, and halogens were used in the reaction with acetoacetanilide and 3-amino-1,2,4-triazole under the optimized reaction conditions for the preparation of [1,2,4]triazolo[1,5-*a*]pyrimidines (Table 2). As shown in Table 2, some aryl aldehydes containing various benzyloxy substitutions on their rings were successfully used in this reaction for the preparation of some new [1,2,4]triazolo[1,5-*a*]pyrimidines, which were not reported at all. As depicted from the results in Scheme 3, [CuFe_2_O_4_@SiO_2_/propyl-1-(*O*-vanillinaldimine)][ZnCl_2_] was successfully used in the reaction to give the products in high yields and short reaction times.

**Table 2:** The preparation of [1,2,4]triazolo[1,5-*a*]pyrimidines

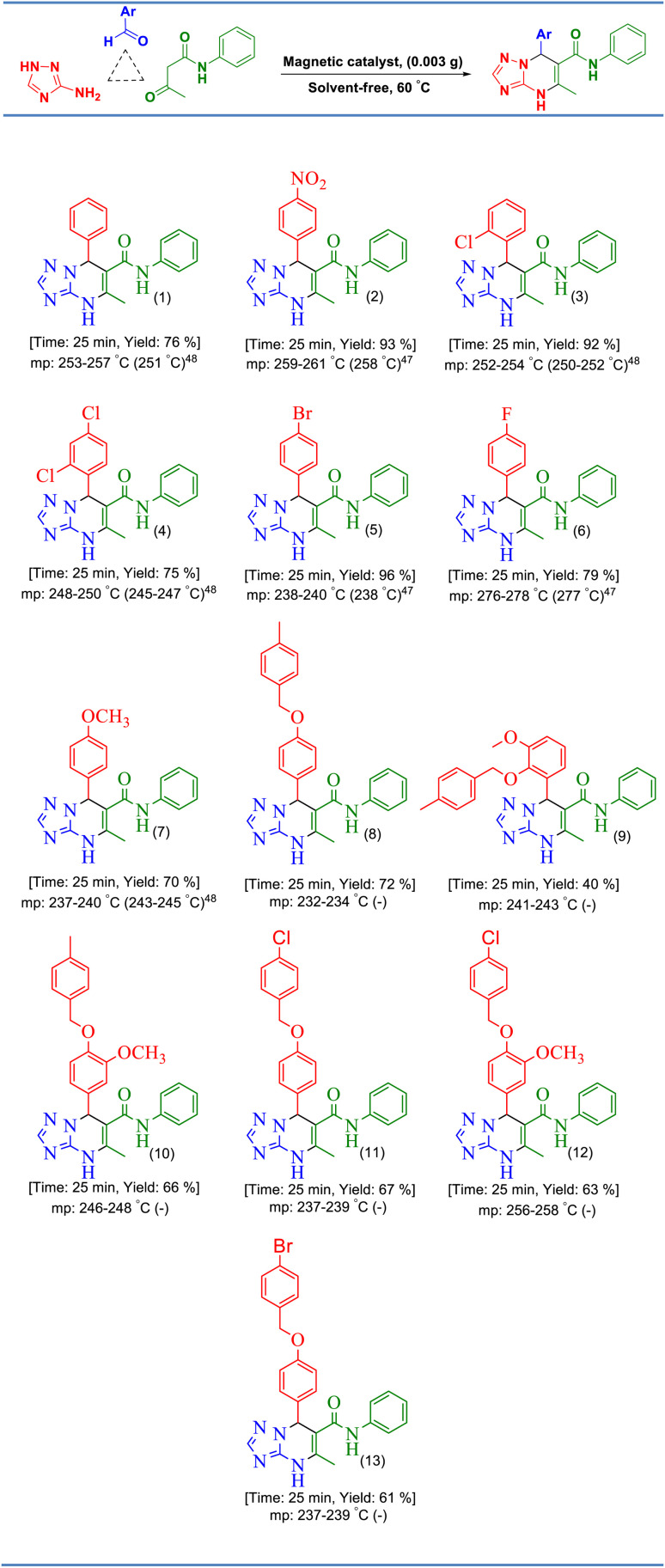

**Scheme 4:**
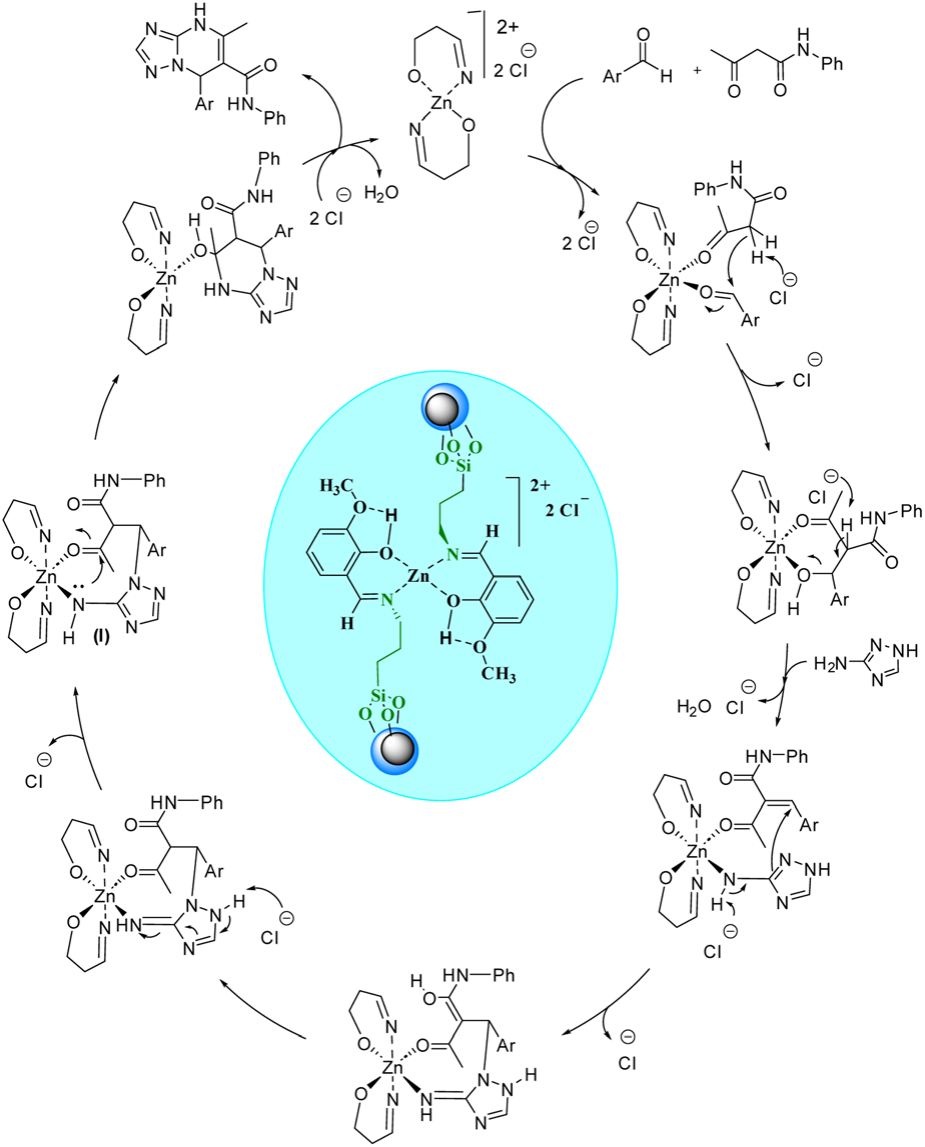
The proposed mechanism for the synthesis of [1,2,4]triazolo[1,5-*a*]pyrimidines.

Regarding the proposed mechanism of this reaction, it should be mentioned that due to the presence of bulky groups on nitrogen atoms in the structure of the catalyst, the zinc complex can change from a tetrahedral structure to a square planar structure to reduce repulsion (Scheme 3). Then, the zinc metal accepts acetoacetanilide and aldehyde compounds as ligands in the cis position relative to each other. In the next step, the chloride anion in the reaction medium causes the Knoevenagel condensation reaction by separating the alpha hydrogen from the acetoacetanilide compound, and the cyanoolefin derivative is formed. With the addition of aldehyde to acetoacetanilide and the removal of one molecule of water, a vacant coordination space is created that can be occupied by 3- amino-1,2,4-triazole. With the participation of the chloride anion, 3-amino-1,2,4-triazole and the cyanoolefin derivative undergo the aza Michael reaction resulting in intermediate (I). Then, with the intramolecular nucleophilic attack in this intermediate, the desired product is formed and separated from the zinc complex. Finally, the chloride anions in the reaction media return to the complex structure (Scheme 4).

### 3.4. Efficiency of catalyst

**Fig. 8:**
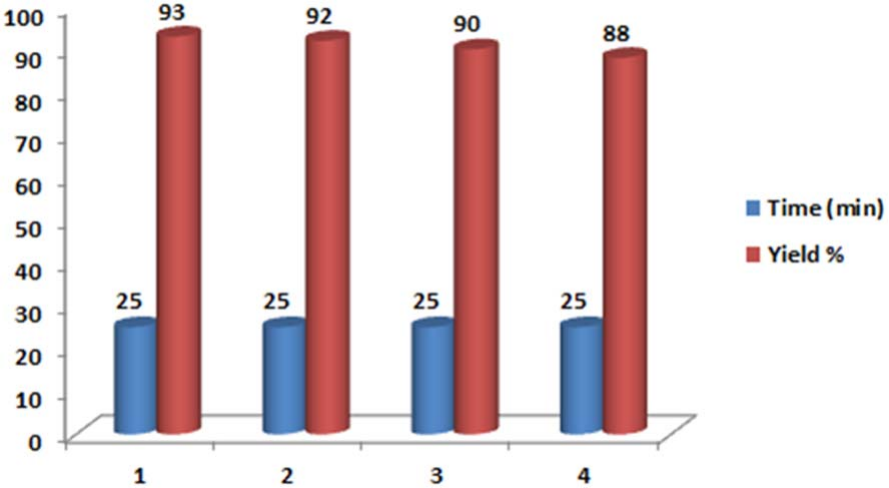
The recovery of nano magnetite Schiff base complex.

One of the important advantages of magnetic catalysts is their reusability in chemical reactions. For this purpose, the recovery of the catalyst in the model reaction was studied. In this study, after the completion of the reaction, the reaction mixture was extracted using hot ethanol and separated from the catalyst. The isolated catalyst was reused in another reaction after washing with acetone. This catalyst was reused three times without a significant decrease in reaction efficiency (Fig. 8).

To find the reason for the decrease in the product yield after the reuse of catalyst, the leaching of zinc on the catalyst surface was studied by atomic absorption spectroscopy. According to this study, zinc constituted 1.38% of the fresh catalyst and there was a 0.64% gain in the amount of zinc on the reused catalyst after four runs. Therefore, one of the reasons for the decrease of yield of product can be attributed to leaching of zinc onto the catalyst surface.

To show the superiority of the new method over the previous methods, the results obtained with the nano magnetite Schiff base complex as a catalyst were compared with some catalysts used in the reaction of 4-nitrobenzaldehyde with acetoacetanilide and 3-amino-1,2,4-triazole. In the third method, acetoacetanilide was *in situ* prepared in the reaction. As can be summarized from Table 3, the presented method is superior to the other catalysts in terms of the isolated yield of the related product and reusability of the catalyst.

**Table 3:** The comparison of the presented catalyst to some other catalysts on the reaction of 4-nitrobenzaldehyde with acetoacetanilide and 3-amino-1,2,4-triazole

Catalyst (amount)	Condition	Time (min)	Yield (%)	Ref.
Maltose (25 mol%)	Solvent free, 80 °C	25	90	47
TSAT (10 mol%)	Solvent free, 60 °C	10	90	48
*p*-TsOH (10 mol%)	H_2_O, reflux	240	87	45
Nano magnetic catalyst (3 mg)	Solvent free, 60 °C	25	93	Our work

## 4 Conclusions

In summary, we have prepared and fully characterized [CuFe_2_O_4_@SiO_2_/propyl-1-(*O*-vanillinaldimine)][ZnCl_2_] by various methods and then successfully tested it as a reusable, heterogeneous and supported magnetite catalyst for the one-pot multi-component reaction of various aryl aldehydes with acetoacetanilide and 3-amino-1,2,4-triazole for the preparation of [1,2,4]triazolo[1,5-*a*]pyrimidines. Some of the products containing the benzyloxy moiety in their structures are new and are being reported for the first time.

## Conflicts of interest

We thank the Hamedan University of Technology for financial support to our research group.

## Supplementary Material

RA-014-D4RA02339K-s001
