# Supplementary material for: The preparation of [1,2,4]triazolo[1,5-a]pyrimidines catalyzed by Schiff base zinc(ii) complex supported on magnetite nanoparticles under mild conditions
Source: RSC Adv. 2024 Jun 14;14(27):19167–73. doi: 10.1039/d4ra02339k (PMC11177290; doi:10.1039/d4ra02339k)
Supplement: RA-014-D4RA02339K-s001 [file RA-014-D4RA02339K-s001.pdf]

Electronic Supplementary Material (ESI) for RSC Advances.
This journal is © The Royal Society of Chemistry 2024

Supporting Information

The preparation of [1,2,4]Triazolo[1,5-*a*]pyrimidines catalyzed by Schiff base zinc (II) complex supported on magnetite nanoparticles under mild condition

Ahmad Reza Moosavi-Zare,* Raha Najafi, Hamid Goudarziafshar

[a]Department of Chemical Engineering, Hamedan University of Technology, Hamedan,65155, Iran .

*E-mail: moosavizare@yahoo.com

Spectral data of compounds:

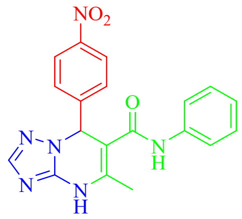

5-methyl-7-(4-nitrophenyl)-N-phenyl-4,7-dihydro-[1,2,4]triazolo[1,5-a]pyrimidine-6-carboxamide

White Solid; M.p: 259-261 °C; IR (KBr, cm^{-1}): 3255, 3101, 2915, 1667, 1621, 1596, 1247, 825, 749, 545; ^{1}H NMR (250 MHz, DMSO-d_6): δ 2.17 (s, 3H, CH_3), 6.66 (s, 1H, H-benzylic), 6.99 (s, 1H), 7.22 (s, 2H, ArH), 7.47 (d, J = 6.25 Hz, 4H, ArH), 7.67 (s, 1H, ArH), 8.16 (d, J = 6.75 Hz, 2H, ArH), 9.78 (s, 1H, NH), 10.39 (s, 1H, NH); ^{13}C-NMR (DMSO-d_6, 62.5 MHz): δ 25.4, 60.0, 103.0, 120.1, 123.8, 124.2, 129.0, 130.0, 137.8, 138.4, 139.2, 147.6, 148.0, 148.3, 149.2, 150.7, 165.0.

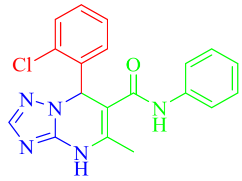

7-(2-chlorophenyl)-5-methyl-N-phenyl-4,7-dihydro-[1,2,4]triazolo[1,5-a]pyrimidine-6-carboxamide

White Solid; M.p: 252-254 °C;IR (KBr, cm^{-1}): 3414, 3299, 3098, 3037, 2866, 1670,1636, 1595, 1499, 1328, 1262, 975, 872,750, 546; ^{1}H NMR (250 MHz, DMSO-d_6): δ 2.14 (s, 3H, CH_3), 6.92 (s, 1H), 6.97 (s. 1H), 7.27 (s, 5H, ArH), 7.44 (s, 3H, ArH), 7.60 (s, 1H, ArH), 9.81 (s, 1H, NH),

10.28 (s, 1H, NH); ^{13}C-NMR (DMSO-d_6, 62.5 MHz): δ 17.5, 58.2, 103.2, 119.9, 123.7, 128.0, 129.0, 130.3, 130.5, 132.5, 137.2, 138.1, 139.3, 148.3, 150.4, 165.0.

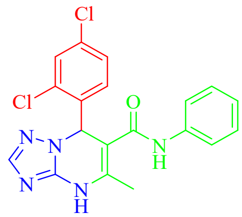

7-(2,4-dichlorophenyl)-5-methyl-N-phenyl-4,7-dihydro-[1,2,4]triazolo[1,5-a]pyrimidine-6-carboxamide

White Solid; M.p: 248-250 °C;IR (KBr, cm^{-1}): 3414, 3304, 3095, 3027, 1739, 1670, 1533, 1491, 1332, 1260, 857, 759, 547; ^{1}H NMR (250 MHz, DMSO-d_6): 2.13 (s, 3H, CH3), 6.88 (s, 1H, H-benzylic), 6.98 (s, 1H, ArH), 7.21 (s, 2H, ArH), 7.30 (d, J =7.30 Hz, 1H, ArH), 7.36 (s, 1H, ArH), 7.45 (s, 3H, ArH), 7.52 (s, 1H, ArH), 7.61 (s, 1H, ArH), 9.82 (s, 1H, NH), 10.33 (s, 1H, NH); ^{13}C NMR (DMSO-d_6, 62.5 MHz): δ 17.6, 57.8, 59.1, 102.7, 119.9, 123.8, 128.3, 129.0, 129.5, 131.9, 133.6, 134.0, 137.5, 139.3, 148.3, 150.6, 164.9.

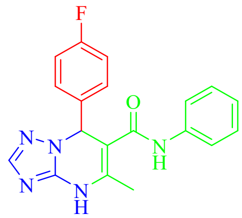

7-(4-fluorophenyl)-5-methyl-N-phenyl-4,7-dihydro-[1,2,4]triazolo[1,5-a]pyrimidine-6-carboxamide

White Solid; M.p: 276-278 °C;IR (KBr, cm^{-1}): 3270, 3102, 2977, 1663, 1627, 1595, 1509, 1325, 1231, 977, 839, 747, 516; ^{1}H NMR (250 MHz, DMSO-d_6): δ 2.16 (s, 3H, CH3), 6.53 (s, 1H), 6.95-7.01 (m, 1H), 7.10 (d, J = 8.00 Hz, 2H, ArH), 7.15 (s, 1H), 7.24 (d, J = 6.75 Hz, 3H, ArH), 7.48 (d, J = 7.5 Hz, 2H, ArH), 7.63 (s, 1H, ArH), 9.73 (s, 1H, NH), 10.24 (s, 1H, NH); ^{13}C-NMR

(DMSO-d_6, 62.5 MHz): δ 17.7, 60.0, 103.8, 115.5, 115.9, 120.0, 123.7, 129.0, 129.7, 137.1, 137.4, 139.3, 148.2, 150.4, 165.2.

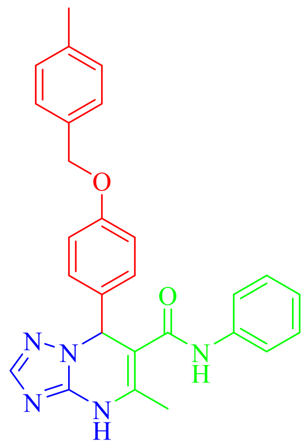

5-methyl-7-(4-((4-methylbenzyl)oxy)phenyl)-N-phenyl-4,7-dihydro-[1,2,4]triazolo[1,5-a]pyrimidine-6-carboxamide

White Solid; M.p: 232-234°C; IR (KBr, cm^{-1}): 3445, 3271, 3101, 3027, 2920, 2869, 1667, 1626, 1596,1510, 1441, 1330, 1243, 1010, 750, 728, 691 ; ^{1}H NMR (250 MHz, DMSO-d_6): δ 2.16 (s, 3H, CH_3), 2.26 (s, 3H, CH_3), 4.95 (s, 2H), 6.47 (s, 1H), 6.89 (d, J = 7.00 Hz, 2H, ArH), 7.00 (d, J = 6.50 Hz, 2H, ArH), 7.14 (d, J = 5.75 Hz, 2H, ArH), 7.19 (s, 1H, ArH), 7.24 (s, 3H, ArH), 7.49 (d, J = 6.75 Hz, 3H, ArH), 7.60 (s, 1H, ArH), 9.70 (s, 1H, NH), 10.15 (s, 1H, NH); ^{13}C-NMR (DMSO-d_6, 62.5 MHz): δ 17.7, 21.1, 60.1, 69.5, 104.1, 115.0, 120.0, 123.7, 128.2, 128.8, 129.0, 129.3, 133.4, 134.3, 136.8, 137.5, 139.4, 150.2, 158.6, 165.4; MS: m/z = 451.

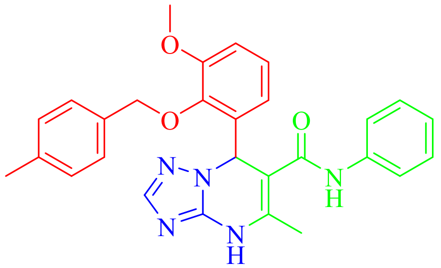

7-(3-methoxy-2-((4-methylbenzyl)oxy)phenyl)-5-methyl-N-phenyl-4,7-dihydro-[1,2,4]triazolo[1,5-a]pyrimidine-6-carboxamide

White Solid; M.p: 241-243°C;IR (KBr, cm^{-1}): 3407, 3274, 3134, 3055, 2890, 1595, 1670, 1589, 1529, 1481, 1314, 1224, 1070, 766 ; ^{1}H NMR (250 MHz, DMSO-d_6): δ 2.12 (s, 3H, CH_3), 2.30 (s, 3H, CH_3), 3.76 (s, 3H, CH_3), 4.73 (d, J = 10.25 Hz, 1H, ArH), 5.01 (d, J = 10.25 Hz, 1H), 6.78-6.84 (m, 2H, ArH), 6.95 (s, 2H, ArH), 7.00 (s, 1H, ArH), 7.18 (s, 3H, ArH), 7.38 (d, J = 7.00 Hz, 2H, ArH), 7.46 (d, J = 7.50 Hz, 2H, ArH), 7.56 (s, 1H, ArH), 9.72 (s, 1H, NH), 10.09 (s, 1H, NH); ^{13}C-NMR (DMSO-d_6, 62.5 MHz): δ 17.6, 21.2, 56.1, 74.4, 104.2, 112.9, 119.8, 120.6, 123.6, 124.7, 128.5, 128.9, 129.1, 134.7, 135.3, 136.5, 137.3, 139.4, 150.1, 152.7. MS: m/z = 481.5.

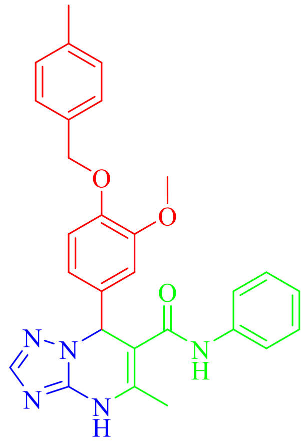

7-(3-methoxy-4-((4-methylbenzyl)oxy)phenyl)-5-methyl-N-phenyl-4,7-dihydro-[1,2,4]triazolo[1,5-a]pyrimidine-6-carboxamide

White Solid; M. p: 246-248°C;IR (KBr, cm^{-1}): 3277, 3098, 3027, 2918, 1673, 1628, 1596, 1515, 1441, 1262, 1003, 754 ; ^{1}H NMR (250 MHz, DMSO-d_6): δ 2.15 (s, 3H, CH_3), 2.26 (s, 3H, CH_3), 3.62 (s, 3H, CH_3), 4.93 (s, 2H), 6.45 (s, 1H), 6.68 (d, J = 8.00 Hz, 1H, ArH), 6.81 (s, 1H, ArH), 6.90-7.02 (m, 2H, ArH), 7.13 (d, J = 7.50 Hz, 2H, ArH), 7.20 (s, 1H, ArH), 7.25 (d, J = 7.50 Hz,

2H, ArH), 7.34 (s, 1H, ArH), 7.50 (d, J = 7.75 Hz, 2H, ArH), 7.62 (s, 1H, ArH), 9.70 (s, 1H, NH), 10.15 (s, 1H, NH); ^{13}C-NMR (DMSO-d_6, 62.5 MHz): δ 17.7, 21.2, 56.0, 60.3, 70.2, 104.0, 111.6, 113.7, 120.0, 123.7, 128.3, 129.0, 129.3, 133.8, 134.4, 136.8, 137.5, 139.4, 148.1, 149.2, 150.2, 165.4; MS: m/z = 481.6.

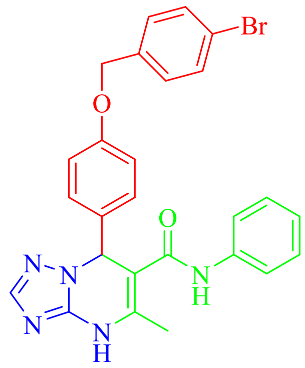

7-(4-((4-bromobenzyl)oxy)phenyl)-5-methyl-N-phenyl-4,7-dihydro-[1,2,4]triazolo[1,5-a]pyrimidine-6-carboxamide

White Solid; M.p: 237-239°C; IR (KBr, cm^{-1}): 3406, 3262, 3103, 2925, 1754, 1665, 1626, 1595, 1509, 1440, 1321, 1242, 1011, 752 ; ^{1}H NMR (250 MHz, DMSO-d_6): δ 2.15 (s, 3H, CH_3), 4.99 (s, 2H), 6.47 (s, 1H), 6.91 (s, 2H, ArH), 6.98 (s, 2H, ArH), 7.14 (d, J = 7.00 Hz, 2H, ArH), 7.22 (s, 2H, ArH), 7.35 (s, 3H, ArH), 7.51-7.59 (m, 4H, ArH), 9.69 (s, 1H, NH), 10.15 (s, 1H, NH); ^{13}C-NMR (DMSO-d_6, 62.5 MHz): δ 17.7, 60.1, 68.8, 104.1, 115.0, 120.0, 121.3, 123.7, 129.0, 130.2, 131.7, 133.6, 136.8, 139.4, 158.3, 165.4.

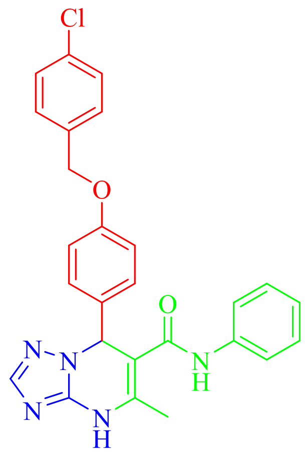

7-(4-((4-chlorobenzyl)oxy)phenyl)-5-methyl-N-phenyl-4,7-dihydro-[1,2,4]triazolo[1,5-a]pyrimidine-6-carboxamide

White Solid; M.p: 237-239 °C; IR (KBr, cm^{-1}): 3263, 3101, 3032, 2925, 1665, 1626, 1595, 1510, 1440, 1329, 1249, 1014, 752; ^{1}H NMR (250 MHz, DMSO-d_6): δ 2.15 (s, 3H, CH_3), 5.01 (s, 2H), 6.47 (s, 1H), 6.91 (d, J = 7.50 Hz, 2H, ArH), 6.98 (s, 1H, ArH), 7.15 (d, J = 7.75 Hz, 2H, ArH), 7.21 (d, J = 7.25 Hz, 2H, ArH), 7.40 (s, 4H, ArH), 7.49 (d, J = 7.25 Hz, 2H, ArH), 7.60 (s, 1H, ArH), 9.69 (s, 1H, NH), 10.15 (s, 1H, NH); ^{13}C-NMR (DMSO-d_6, 62.5 MHz): δ 17.7, 60.0, 68.7, 115.0, 119.9, 123.7, 128.8, 129.9, 133.6, 139.4, 150.2, 158.4, 165.4; MS: m/z = 477.4.

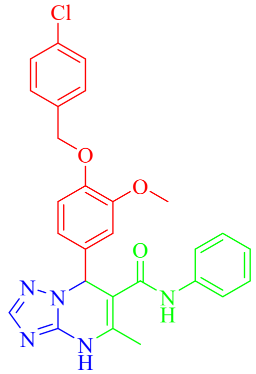

7-(4-((4-chlorobenzyl)oxy)-3-methoxyphenyl)-5-methyl-N-phenyl-4,7-dihydro-[1,2,4]triazolo[1,5-a]pyrimidine-6-carboxamide

White Solid; M.p: 256-258 °C; IR (KBr, cm^{-1}): 3263, 3103, 2917, 2865, 1667, 1626, 1509, 1596, 1515, 1440, 1330, 1228, 1031, 874, 753, 541; ^{1}H NMR (250 MHz, DMSO-d_6): δ 2.15 (s, 3H, CH_3), 3.63 (s, 3H, CH_3), 4.98 (s, 2H), 6.46 (s, 1H), 6.69 (d, J = 7.00 Hz, 1H, ArH), 6.82 (s, 1H, ArH), 6.90-6.98 (m, 2H, ArH), 7.23 (s, 2H, ArH), 7.39 (s, 4H, ArH), 7.50 (d, J = 6.75 Hz, 2H, ArH), 7.62 (s, 1H, ArH), 9.70 (s, 1H, NH), 10.15 (s, 1H, NH); ^{13}C-NMR (DMSO-d_6, 62.5 MHz): δ 17.7, 56.0, 60.3, 69.5, 104.0, 111.7, 113.9, 119.9, 123.7, 128.8, 129.0, 129.9, 132.8, 134.1, 136.5, 136.8, 139.4, 147.9, 149.2, 150.2, 165.4; MS: m/z = 507.5.

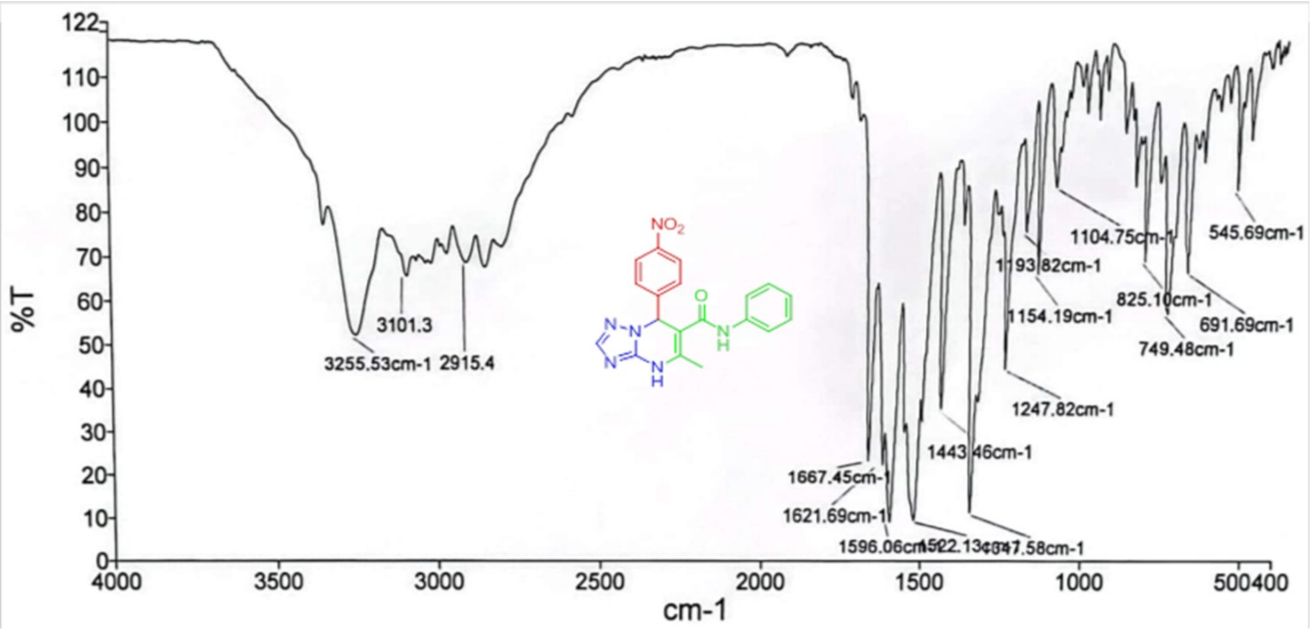
%T
cm-1
3255.53cm-1
3101.3
2915.4
1667.45cm-1
1621.69cm-1
1596.06cm-1
1443.46cm-1
1247.82cm-1
1154.19cm-1
1104.75cm-1
825.10cm-1
749.48cm-1
691.69cm-1
545.69cm-1
NO2

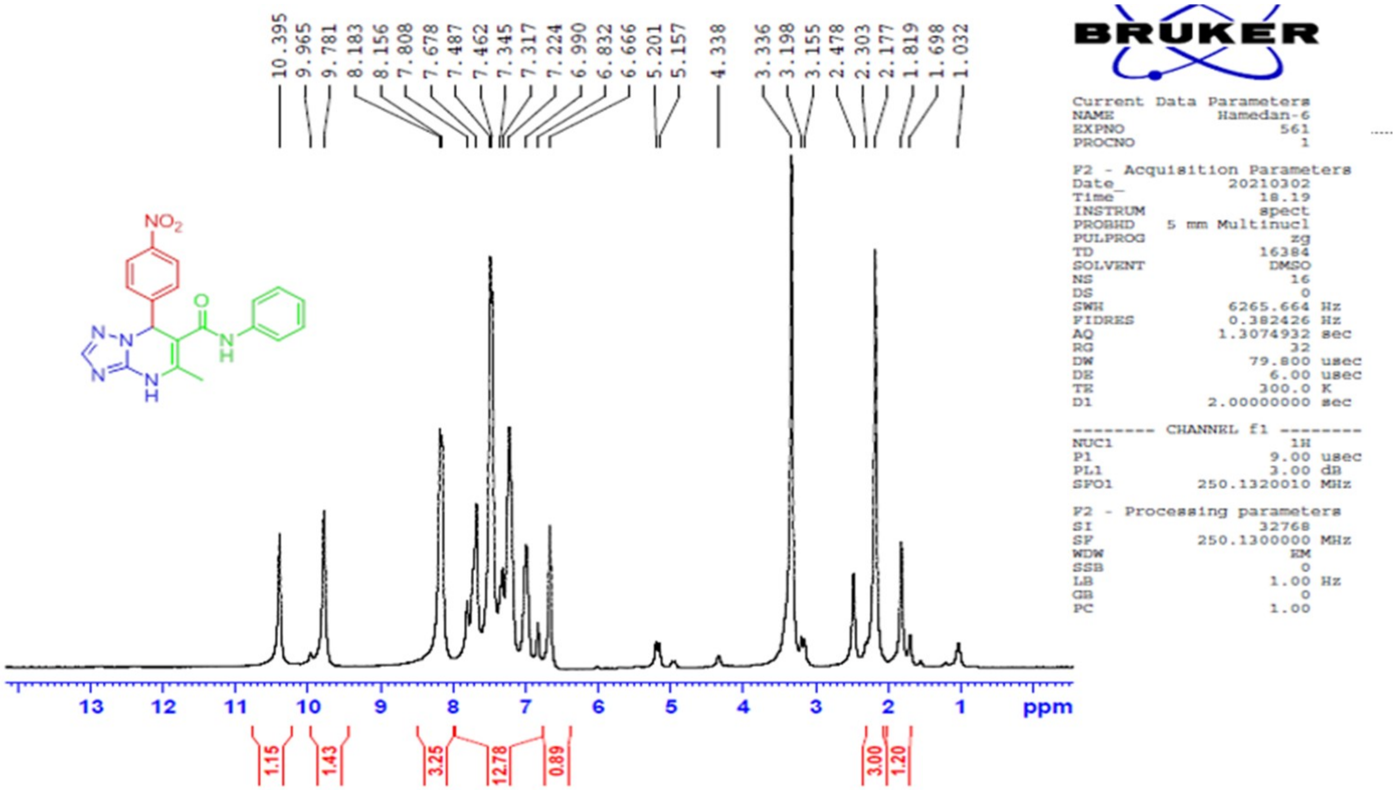

10.395 9.965 9.781 8.183 8.156 7.808 7.678 7.487 7.462 7.345 7.317 7.224 6.990 6.832 6.666 5.201 5.157 4.338 3.336 3.198 3.155 2.478 2.303 2.177 1.819 1.698 1.032
NO2
O
N
H
N
N
N
N
H
13 12 11 10 9 8 7 6 5 4 3 2 1 ppm
1.15 1.43 3.25 12.78 0.89 3.00 1.20
BRUKER
Current Data Parameters
NAME Hamedan-6
EXPNO 561
PROCNO 1
F2 - Acquisition Parameters
Date_ 20210302
Time 18.19
INSTRUM spect
PROBHD 5 mm Multinucl
PULPROG zg
TD 16384
SOLVENT DMSO
NS 16
DS 0
SWH 6265.664 Hz
FIDRES 0.382426 Hz
AQ 1.3074932 sec
RG 32
DW 79.800 usec
DE 6.00 usec
TE 300.0 K
D1 2.00000000 sec
======== CHANNEL f1 ========
NUC1 1H
P1 9.00 usec
PL1 3.00 dB
SFO1 250.1320010 MHz
F2 - Processing parameters
SI 32768
SF 250.1300000 MHz
WDW EM
SSB 0
LB 1.00 Hz
GB 0
PC 1.00

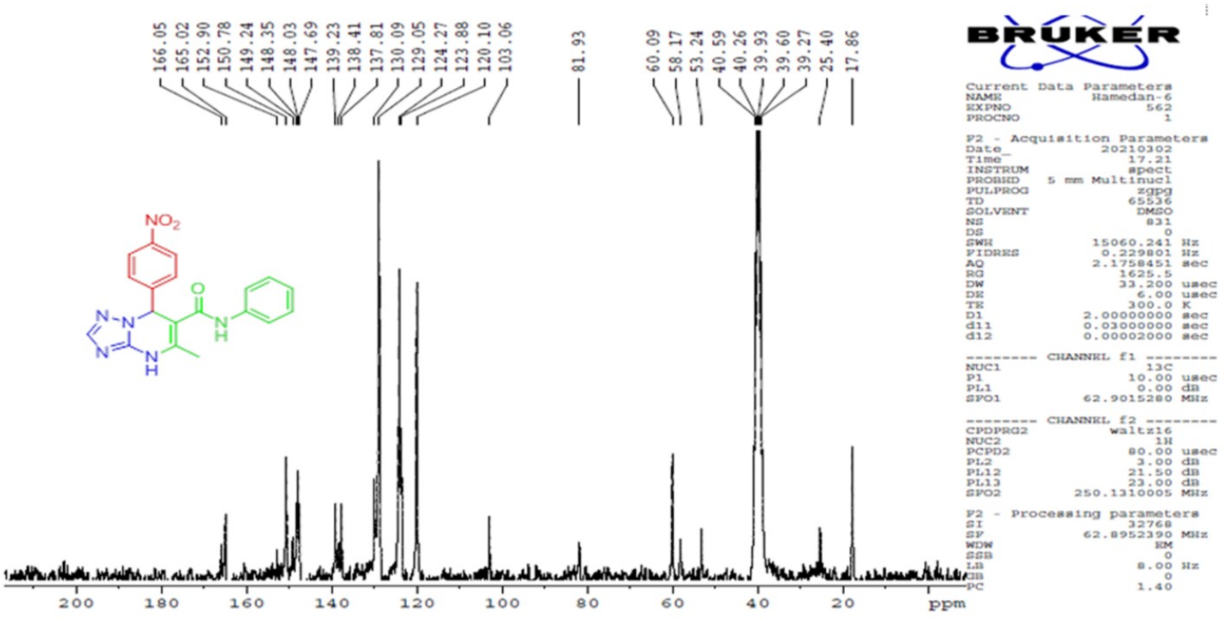

166.05
165.02
152.90
150.78
149.24
148.35
148.03
147.69
139.23
138.41
137.81
130.09
129.05
124.27
123.88
120.10
103.06
81.93
60.09
58.17
53.24
40.59
40.26
39.93
39.60
39.27
25.40
17.86
NO2
O
N
H
N
N
N
N
H
BRUKER
Current Data Parameters
NAME Hamedan-6
EXPNO 562
PROCNO 1
F2 - Acquisition Parameters
Date_ 20210302
Time 17.21
INSTRUM spect
PROBHD 5 mm Multinucl
PULPROG zgpg
TD 65536
SOLVENT DMSO
NS 831
DS 0
SWH 15060.241 Hz
FIDRES 0.229801 Hz
AQ 2.1758451 sec
RG 1625.5
DW 33.200 usec
DE 6.00 usec
TE 300.0 K
D1 2.00000000 sec
d11 0.03000000 sec
d12 0.00002000 sec
======== CHANNEL f1 ========
NUC1 13C
P1 10.00 usec
PL1 0.00 dB
SFO1 62.9015280 MHz
======== CHANNEL f2 ========
CPDPRG2 waltz16
NUC2 1H
PCPD2 80.00 usec
PL2 3.00 dB
PL12 21.50 dB
PL13 23.00 dB
SFO2 250.1310005 MHz
F2 - Processing parameters
SI 32768
SF 62.8952390 MHz
WDW EM
SSB 0
LB 8.00 Hz
GB 0
PC 1.40
200 180 160 140 120 100 80 60 40 20 ppm

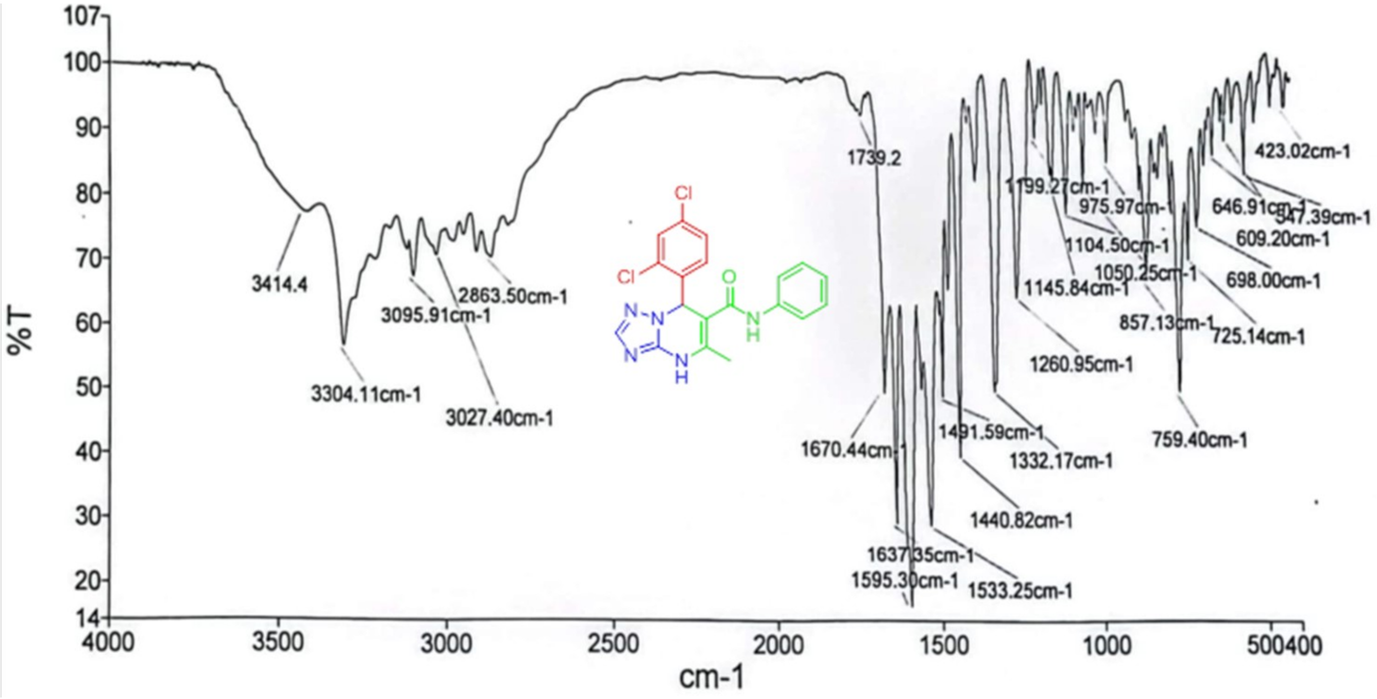
%T
cm-1
3414.4
3304.11cm-1
3095.91cm-1
3027.40cm-1
2863.50cm-1
1739.2
1670.44cm-1
1637.35cm-1
1595.30cm-1
1533.25cm-1
1491.59cm-1
1440.82cm-1
1332.17cm-1
1260.95cm-1
1199.27cm-1
1145.84cm-1
1104.50cm-1
1050.25cm-1
975.97cm-1
857.13cm-1
759.40cm-1
725.14cm-1
698.00cm-1
646.91cm-1
609.20cm-1
547.39cm-1
423.02cm-1

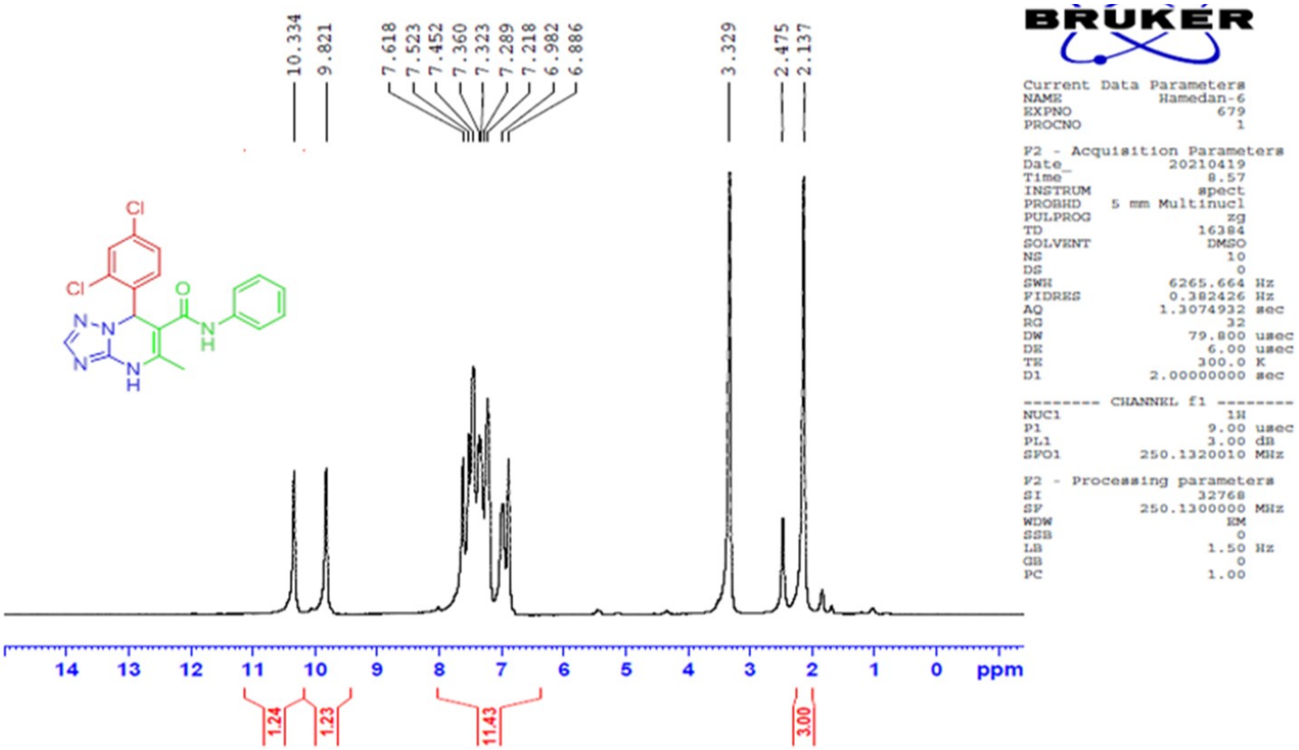

10.334
9.821
7.618
7.523
7.452
7.360
7.323
7.289
7.218
6.982
6.886
3.329
2.475
2.137
Cl
Cl
O
N
H
N
N
N
N
H
14 13 12 11 10 9 8 7 6 5 4 3 2 1 0 ppm
1.24
1.23
11.43
3.00
BRUKER
Current Data Parameters
NAME Hamedan-6
EXPNO 679
PROCNO 1
F2 - Acquisition Parameters
Date_ 20210419
Time 8.57
INSTRUM spect
PROBHD 5 mm Multinucl
PULPROG zg
TD 16384
SOLVENT DMSO
NS 10
DS 0
SWH 6265.664 Hz
FIDRES 0.382426 Hz
AQ 1.3074932 sec
RG 32
DW 79.800 usec
DE 6.00 usec
TE 300.0 K
D1 2.00000000 sec
-------- CHANNEL f1 --------
NUC1 1H
P1 9.00 usec
PL1 3.00 dB
SFO1 250.1320010 MHz
F2 - Processing parameters
SI 32768
SF 250.1300000 MHz
WDW EM
SSB 0
LB 1.50 Hz
GB 0
PC 1.00

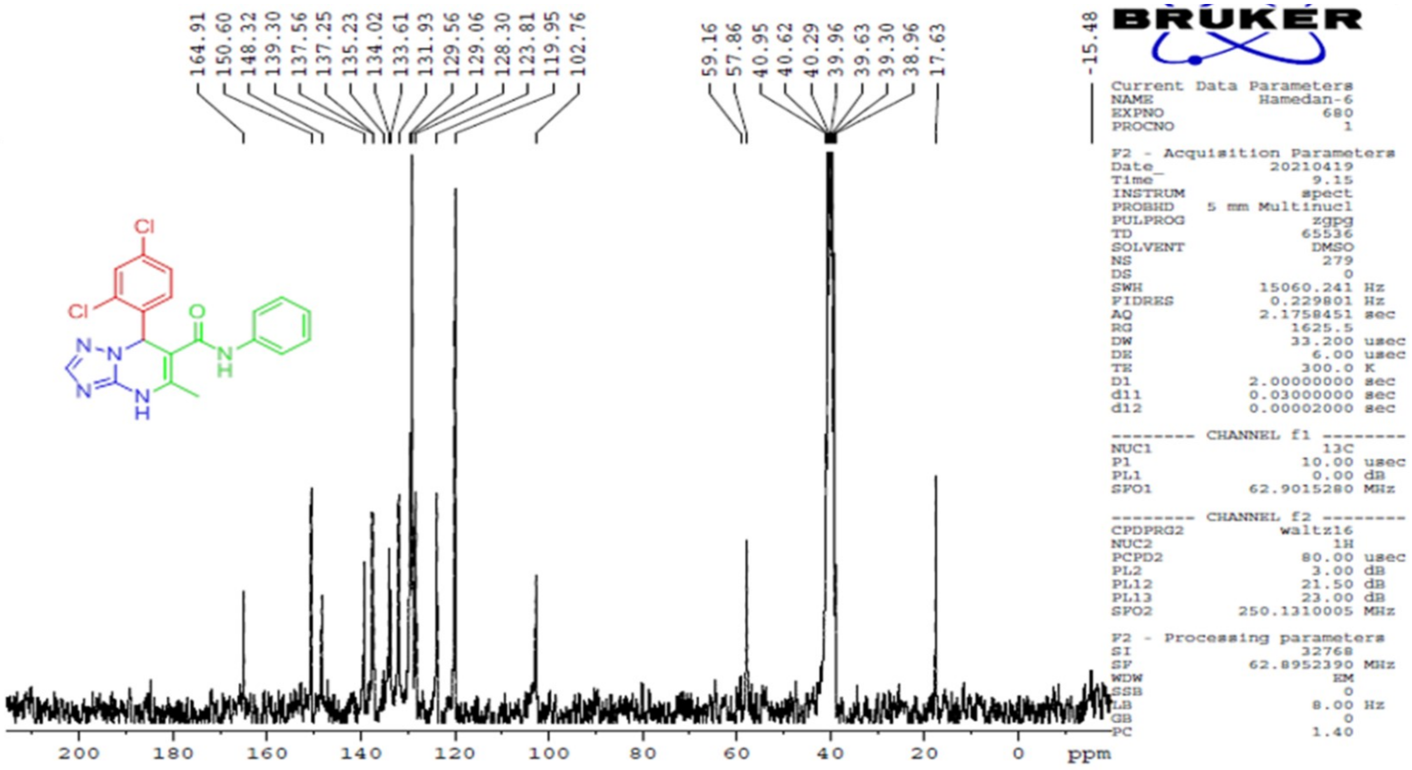
164.91
150.60
148.32
139.30
137.56
137.25
135.23
134.02
133.61
131.93
129.56
129.06
128.30
123.81
119.95
102.76
59.16
57.86
40.95
40.62
40.29
39.96
39.63
39.30
38.96
17.63
-15.48
BRUKER
Current Data Parameters
NAME Hamedan-6
EXPNO 680
PROCNO 1
F2 - Acquisition Parameters
Date_ 20210419
Time 9.15
INSTRUM spect
PROBHD 5 mm Multinucl
PULPROG zgpg
TD 65536
SOLVENT DMSO
NS 279
DS 0
SWH 15060.241 Hz
FIDRES 0.229801 Hz
AQ 2.1758451 sec
RG 1625.5
DW 33.200 usec
DE 6.00 usec
TE 300.0 K
D1 2.00000000 sec
d11 0.03000000 sec
d12 0.00002000 sec
======== CHANNEL f1 ========
NUC1 13C
P1 10.00 usec
PL1 0.00 dB
SFO1 62.9015280 MHz
======== CHANNEL f2 ========
CPDPRG2 waltz16
NUC2 1H
PCPD2 80.00 usec
PL2 3.00 dB
PL12 21.50 dB
PL13 23.00 dB
SFO2 250.1310005 MHz
F2 - Processing parameters
SI 32768
SF 62.8952390 MHz
WDW EM
SSB 0
LB 8.00 Hz
GB 0
PC 1.40
200 180 160 140 120 100 80 60 40 20 0 ppm

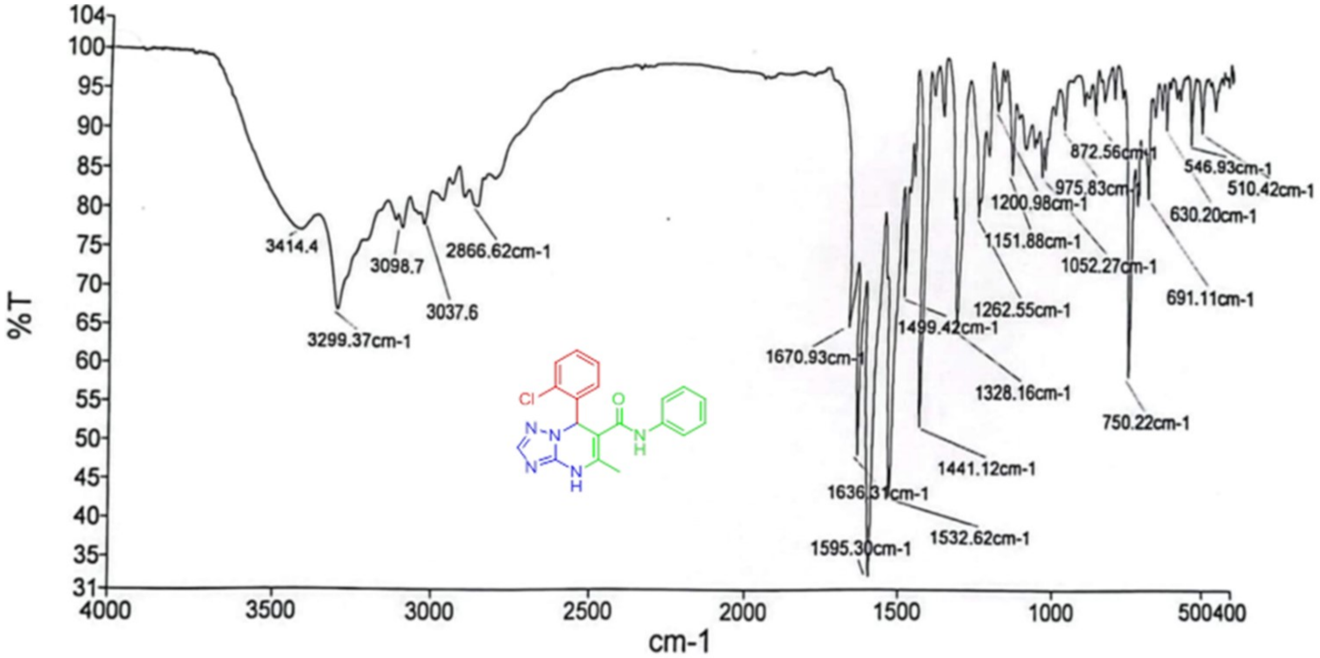
%T
cm-1
3414.4
3299.37cm-1
3098.7
3037.6
2866.62cm-1
1670.93cm-1
1636.31cm-1
1595.30cm-1
1532.62cm-1
1499.42cm-1
1441.12cm-1
1328.16cm-1
1262.55cm-1
1200.98cm-1
1151.88cm-1
1052.27cm-1
975.83cm-1
872.56cm-1
750.22cm-1
691.11cm-1
630.20cm-1
546.93cm-1
510.42cm-1
Cl
O
N
H

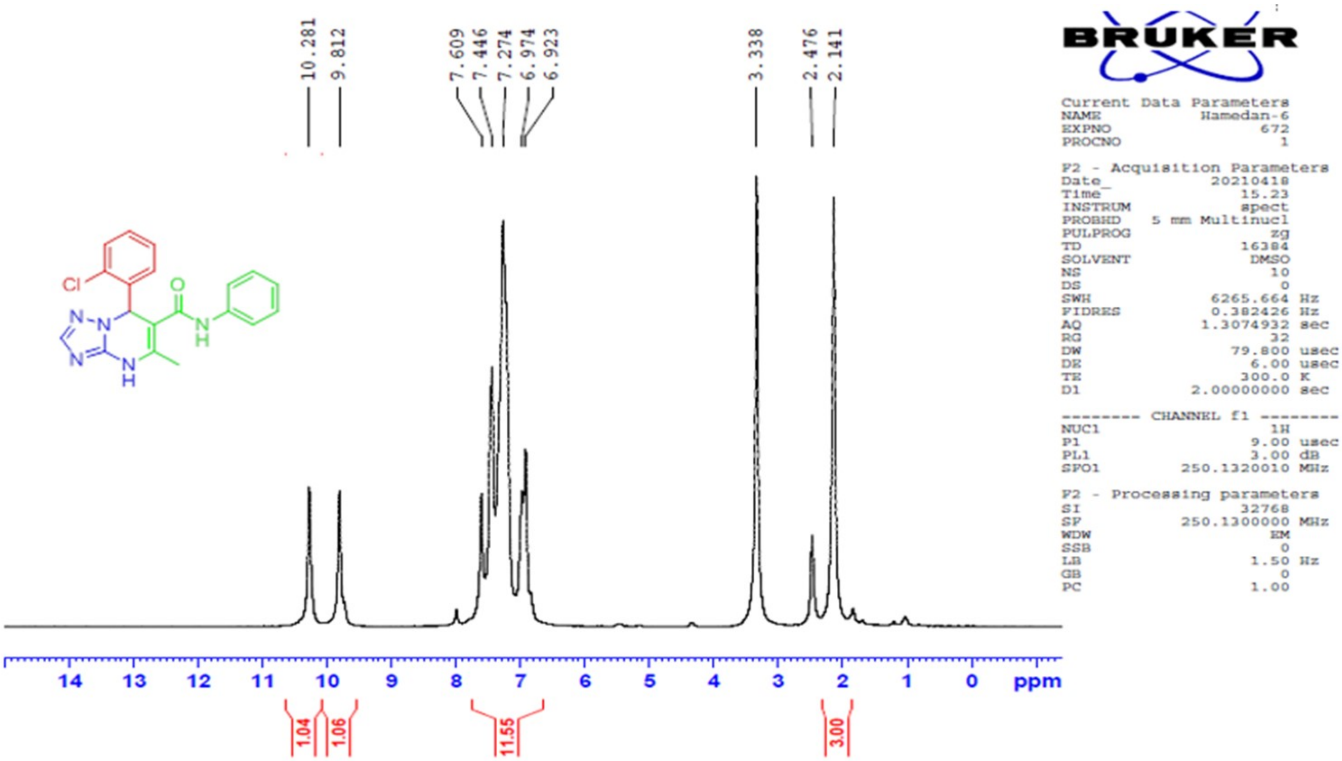
10.281
9.812
7.609
7.446
7.274
6.974
6.923
3.338
2.476
2.141
Cl
O
N
N
N
N
H
H
14 13 12 11 10 9 8 7 6 5 4 3 2 1 0 ppm
1.04
1.06
11.55
3.00
BRUKER
Current Data Parameters
NAME Hamedan-6
EXPNO 672
PROCNO 1
F2 - Acquisition Parameters
Date_ 20210418
Time 15.23
INSTRUM spect
PROBHD 5 mm Multinucl
PULPROG zg
TD 16384
SOLVENT DMSO
NS 10
DS 0
SWH 6265.664 Hz
FIDRES 0.382426 Hz
AQ 1.3074932 sec
RG 32
DW 79.800 usec
DE 6.00 usec
TE 300.0 K
D1 2.00000000 sec
======== CHANNEL f1 ========
NUC1 1H
P1 9.00 usec
PL1 3.00 dB
SFO1 250.1320010 MHz
F2 - Processing parameters
SI 32768
SF 250.1300000 MHz
WDW EM
SSB 0
LB 1.50 Hz
GB 0
PC 1.00

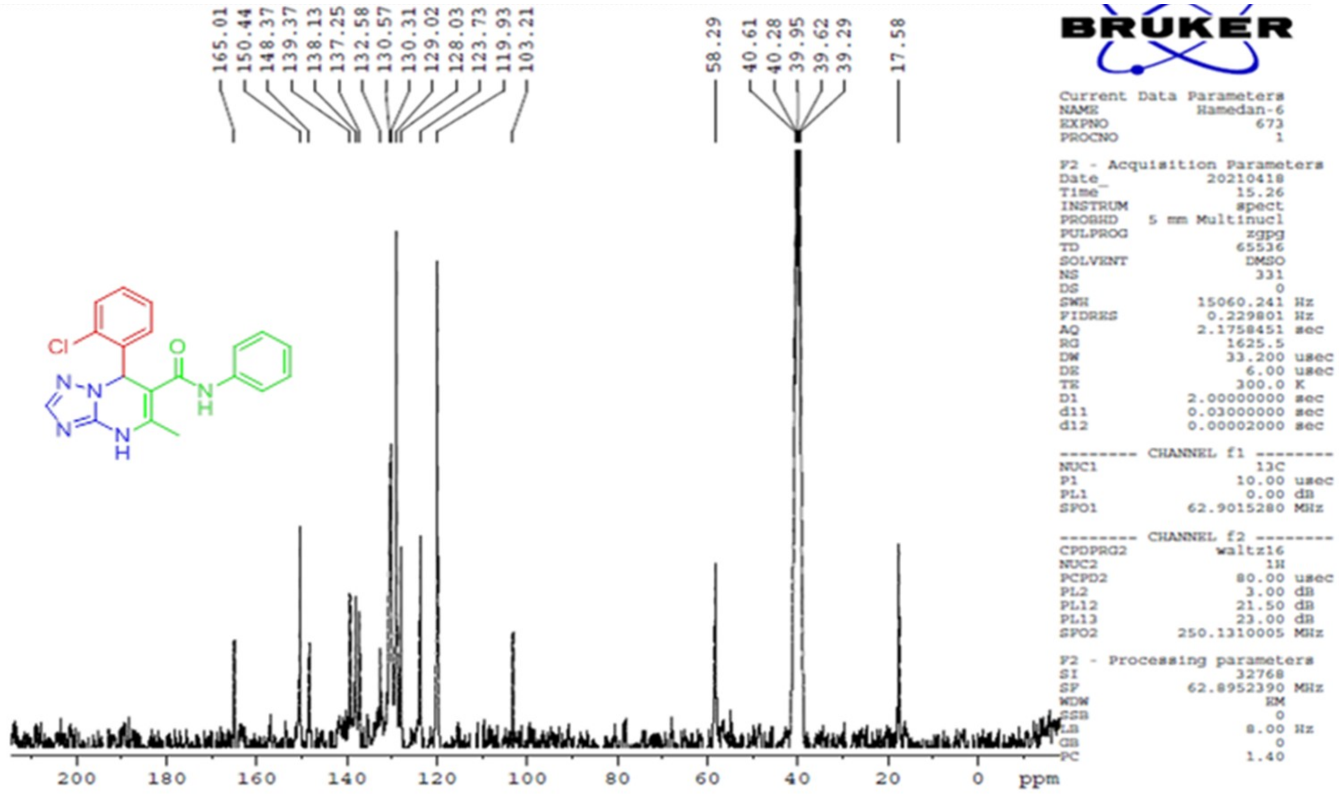

165.01
150.44
148.37
139.37
138.13
137.25
132.58
130.57
130.31
129.02
128.03
123.73
119.93
103.21
58.29
40.61
40.28
39.95
39.62
39.29
17.58
BRUKER
Current Data Parameters
NAME Hamedan-6
EXPNO 673
PROCNO 1
F2 - Acquisition Parameters
Date_ 20210418
Time 15.26
INSTRUM spect
PROBHD 5 mm Multinucl
PULPROG zgpg
TD 65536
SOLVENT DMSO
NS 331
DS 0
SWH 15060.241 Hz
FIDRES 0.229801 Hz
AQ 2.1758451 sec
RG 1625.5
DW 33.200 usec
DE 6.00 usec
TE 300.0 K
D1 2.00000000 sec
d11 0.03000000 sec
d12 0.00002000 sec
======== CHANNEL f1 ========
NUC1 13C
P1 10.00 usec
PL1 0.00 dB
SFO1 62.9015280 MHz
======== CHANNEL f2 ========
CPDPRG2 waltz16
NUC2 1H
PCPD2 80.00 usec
PL2 3.00 dB
PL12 21.50 dB
PL13 23.00 dB
SFO2 250.1310005 MHz
F2 - Processing parameters
SI 32768
SF 62.8952390 MHz
WDW EM
SSB 0
LB 8.00 Hz
GB 0
PC 1.40
200 180 160 140 120 100 80 60 40 20 0 ppm
Cl
O
N
H
N
N
N
N
H

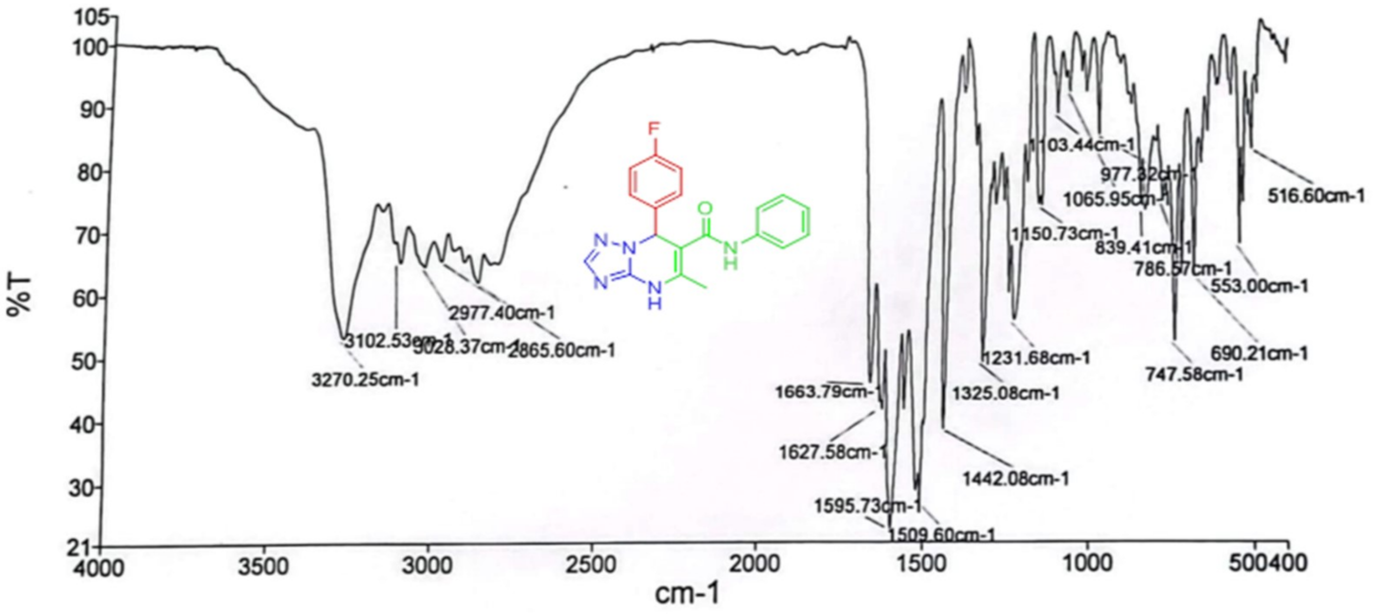

%T
cm-1
3270.25cm-1
3102.53cm-1
3028.37cm-1
2977.40cm-1
2865.60cm-1
1663.79cm-1
1627.58cm-1
1595.73cm-1
1509.60cm-1
1442.08cm-1
1325.08cm-1
1231.68cm-1
1150.73cm-1
1065.95cm-1
977.32cm-1
839.41cm-1
786.57cm-1
747.58cm-1
690.21cm-1
553.00cm-1
516.60cm-1

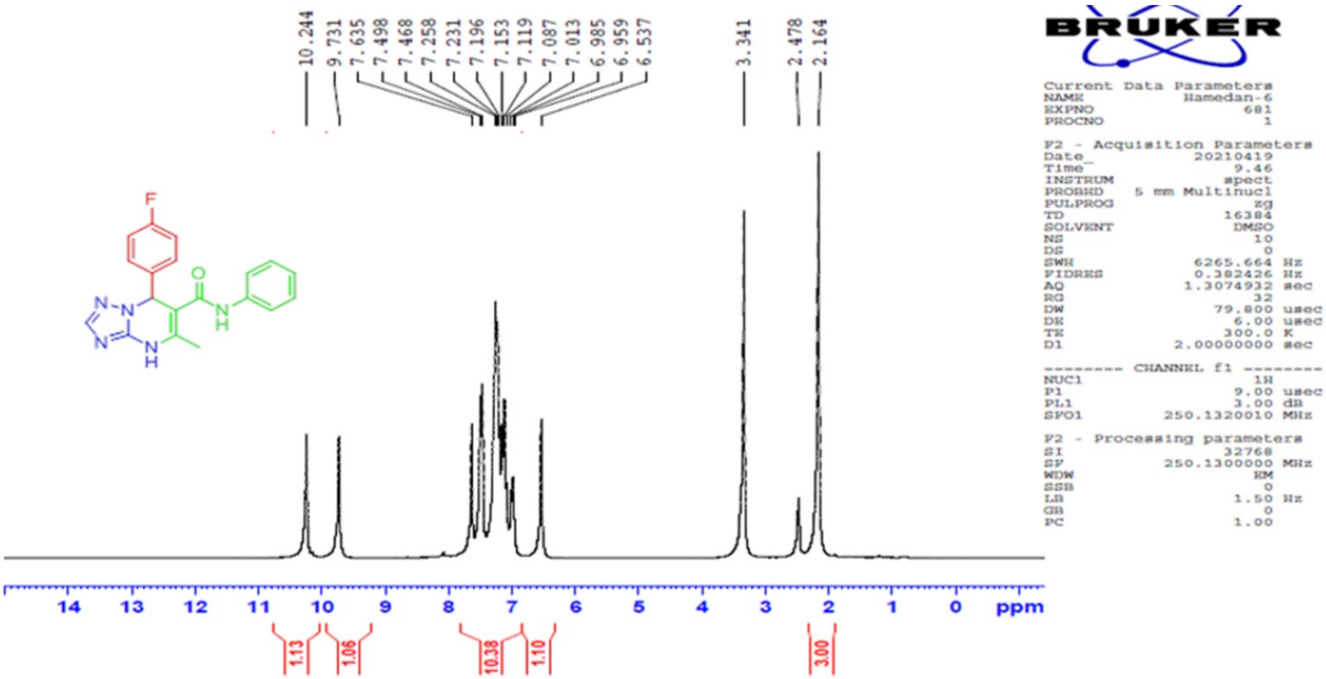

10.244
9.731
7.635
7.498
7.468
7.258
7.231
7.196
7.153
7.119
7.087
7.013
6.985
6.959
6.537
3.341
2.478
2.164
BRUKER
Current Data Parameters
NAME Hamedan-6
EXPNO 681
PROCNO 1
F2 - Acquisition Parameters
Date_ 20210419
Time 9.46
INSTRUM spect
PROBHD 5 mm Multinucl
PULPROG zg
TD 16384
SOLVENT DMSO
NS 10
DS 0
SWH 6265.664 Hz
FIDRES 0.382426 Hz
AQ 1.3074932 sec
RG 32
DW 79.800 usec
DE 6.00 usec
TE 300.0 K
D1 2.00000000 sec
======== CHANNEL f1 ========
NUC1 1H
P1 9.00 usec
PL1 3.00 dB
SFO1 250.1320010 MHz
F2 - Processing parameters
SI 32768
SF 250.1300000 MHz
WDW EM
SSB 0
LB 1.50 Hz
GB 0
PC 1.00
14 13 12 11 10 9 8 7 6 5 4 3 2 1 0 ppm
1.13
1.06
10.38
1.10
3.00

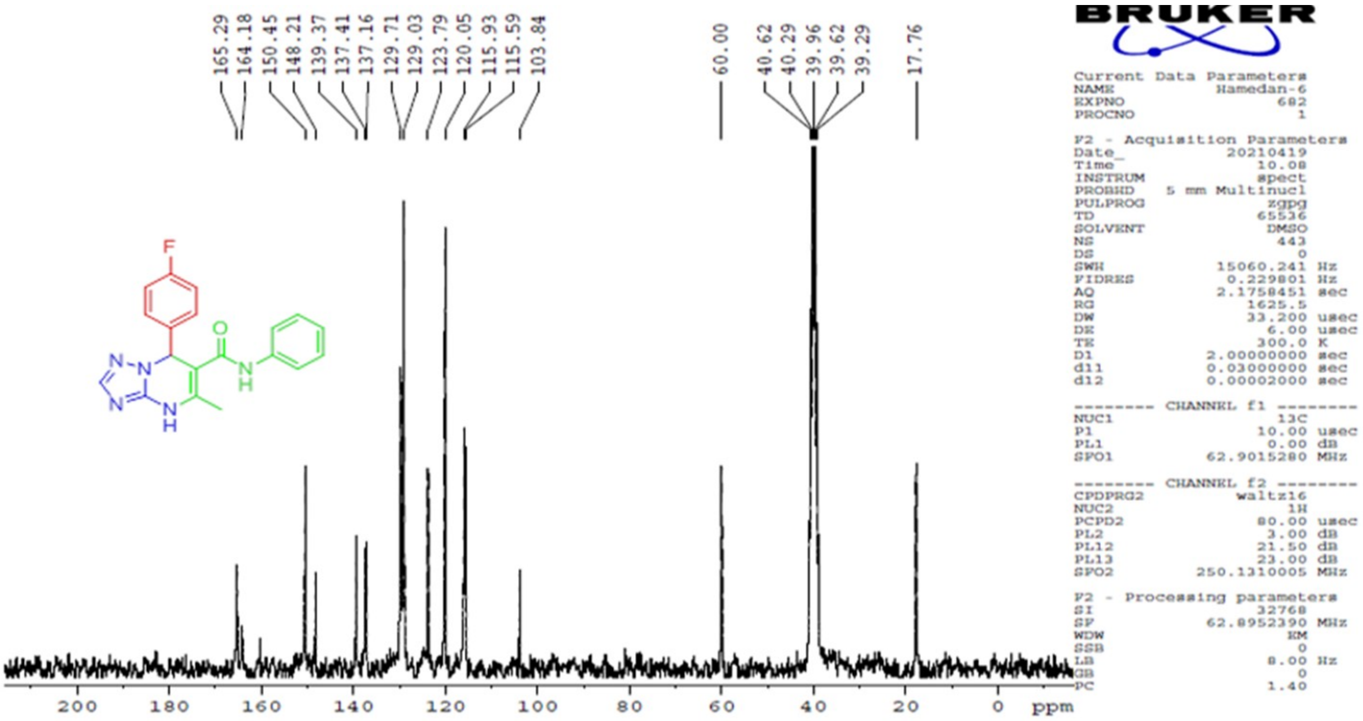
165.29
164.18
150.45
148.21
139.37
137.41
137.16
129.71
129.03
123.79
120.05
115.93
115.59
103.84
60.00
40.62
40.29
39.96
39.62
39.29
17.76
F
N
N
N
N
H
O
N
H
BRUKER
Current Data Parameters
NAME Hamedan-6
EXPNO 682
PROCNO 1
F2 - Acquisition Parameters
Date_ 20210419
Time 10.08
INSTRUM spect
PROBHD 5 mm Multinucl
PULPROG zgpg
TD 65536
SOLVENT DMSO
NS 443
DS 0
SWH 15060.241 Hz
FIDRES 0.229801 Hz
AQ 2.1758451 sec
RG 1625.5
DW 33.200 usec
DE 6.00 usec
TE 300.0 K
D1 2.00000000 sec
d11 0.03000000 sec
d12 0.00002000 sec
======== CHANNEL f1 ========
NUC1 13C
P1 10.00 usec
PL1 0.00 dB
SFO1 62.9015280 MHz
======== CHANNEL f2 ========
CPDPRG2 waltz16
NUC2 1H
PCPD2 80.00 usec
PL2 3.00 dB
PL12 21.50 dB
PL13 23.00 dB
SFO2 250.1310005 MHz
F2 - Processing parameters
SI 32768
SF 62.8952390 MHz
WDW EM
SSB 0
LB 8.00 Hz
GB 0
PC 1.40
200 180 160 140 120 100 80 60 40 20 0 ppm

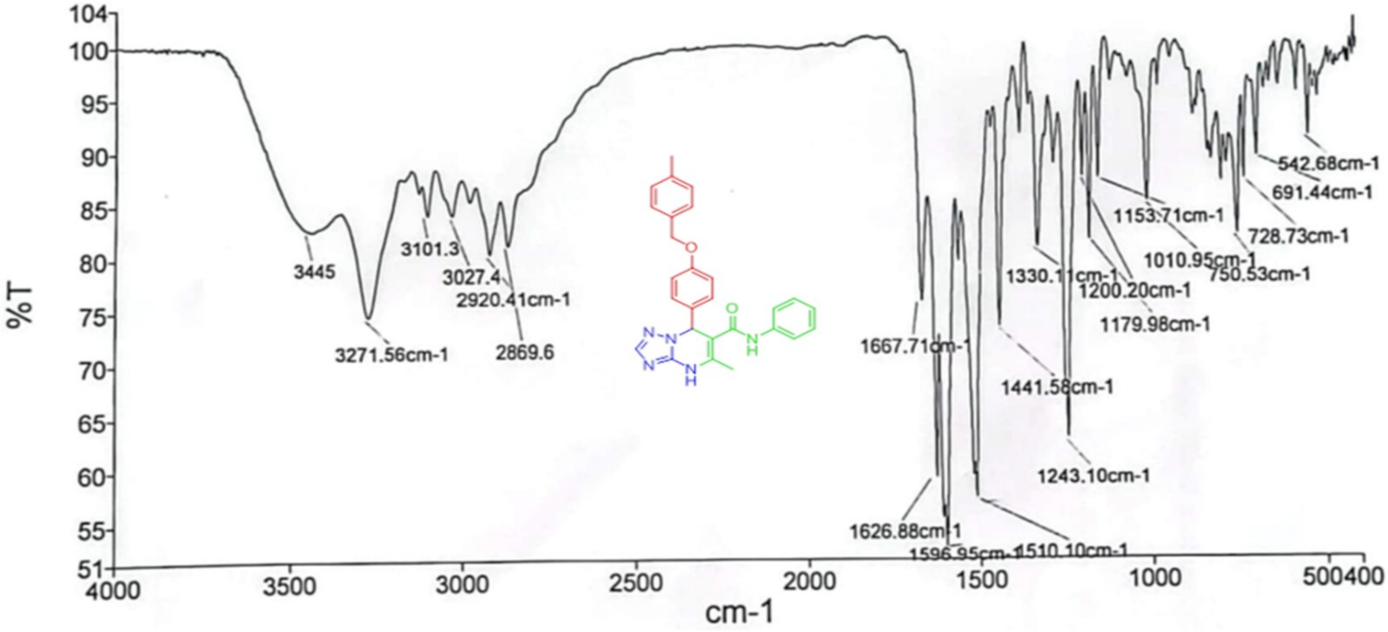

%T
cm-1
3445
3271.56cm-1
3101.3
3027.4
2920.41cm-1
2869.6
1667.71cm-1
1626.88cm-1
1596.95cm-1
1510.10cm-1
1441.58cm-1
1330.11cm-1
1243.10cm-1
1200.20cm-1
1179.98cm-1
1153.71cm-1
1010.95cm-1
750.53cm-1
728.73cm-1
691.44cm-1
542.68cm-1
O
N
H

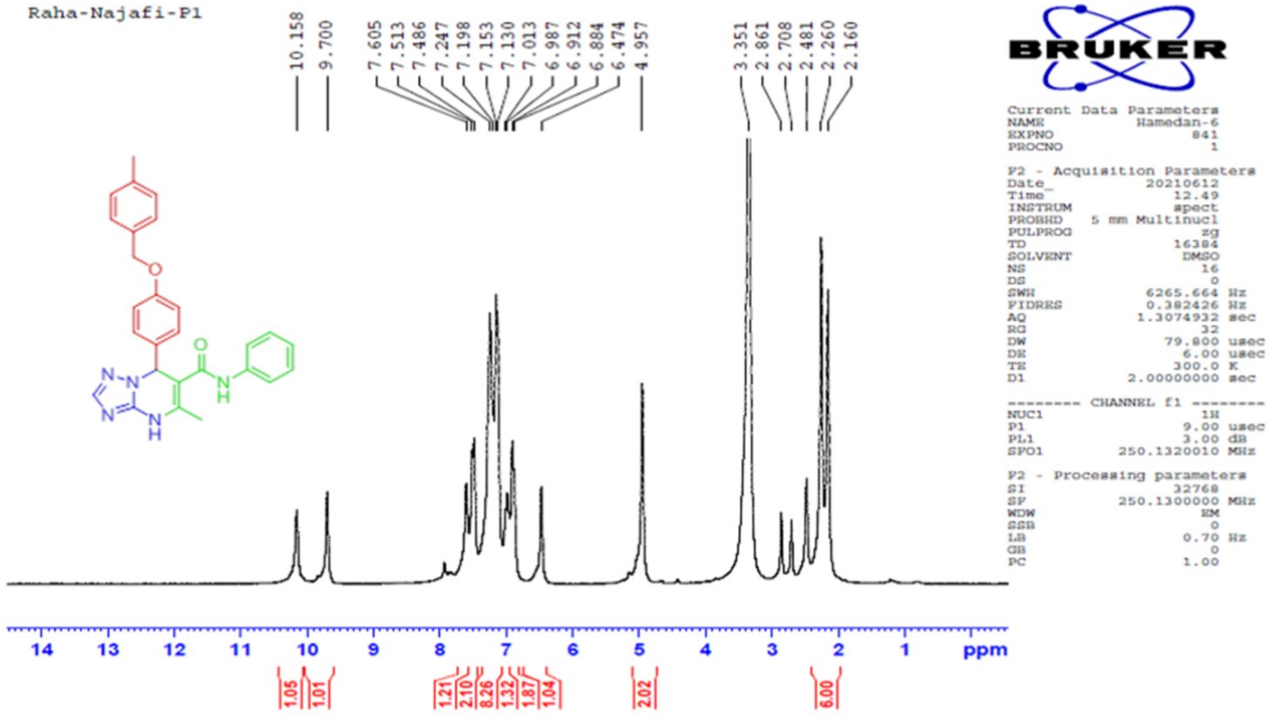
Raha-Najafi-P1
10.158
9.700
7.605
7.513
7.486
7.247
7.198
7.153
7.130
7.013
6.987
6.912
6.884
6.474
4.957
3.351
2.861
2.708
2.481
2.260
2.160
BRUKER
Current Data Parameters
NAME Hamedan-6
EXPNO 841
PROCNO 1
F2 - Acquisition Parameters
Date_ 20210612
Time 12.49
INSTRUM spect
PROBHD 5 mm Multinucl
PULPROG zg
TD 16384
SOLVENT DMSO
NS 16
DS 0
SWH 6265.664 Hz
FIDRES 0.382426 Hz
AQ 1.3074932 sec
RG 32
DW 79.800 usec
DE 6.00 usec
TE 300.0 K
D1 2.00000000 sec
======== CHANNEL f1 ========
NUC1 1H
P1 9.00 usec
PL1 3.00 dB
SFO1 250.1320010 MHz
F2 - Processing parameters
SI 32768
SF 250.1300000 MHz
WDW EM
SSB 0
LB 0.70 Hz
GB 0
PC 1.00
14 13 12 11 10 9 8 7 6 5 4 3 2 1 ppm
1.05
1.01
1.21
2.10
8.26
1.32
1.87
1.04
2.02
6.00

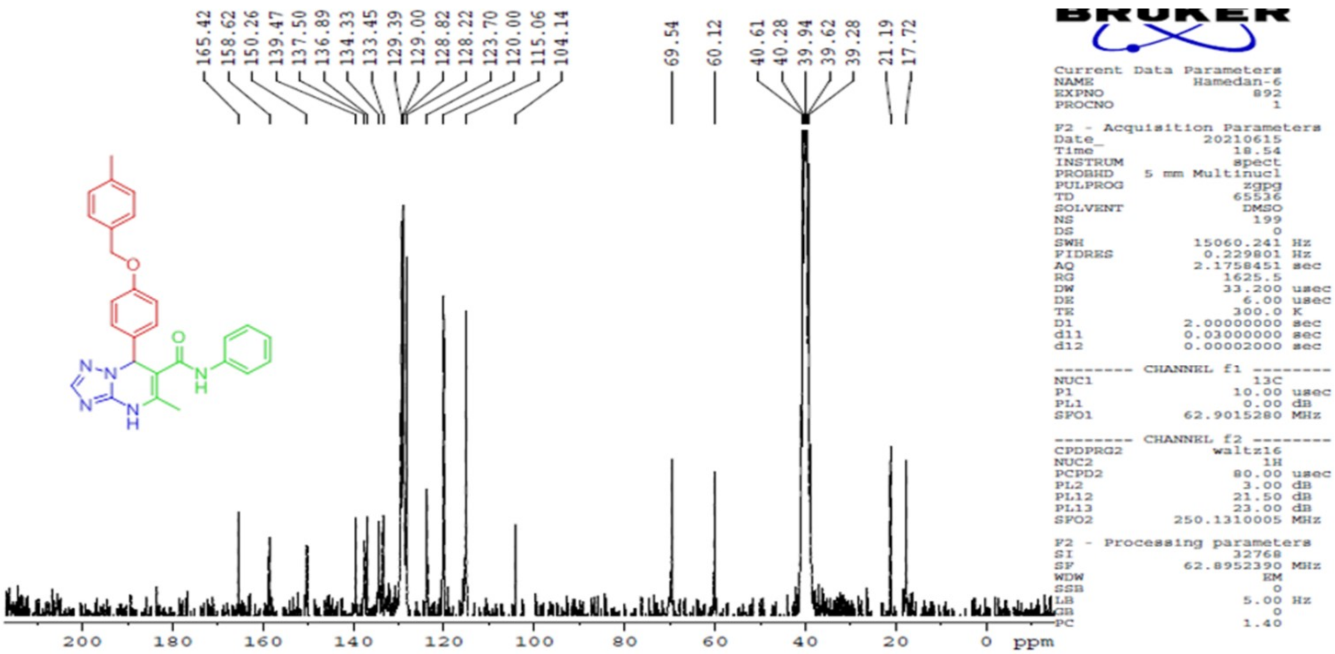
165.42
158.62
150.26
139.47
137.50
136.89
134.33
133.45
129.39
129.00
128.82
128.22
123.70
120.00
115.06
104.14
69.54
60.12
40.61
40.28
39.94
39.62
39.28
21.19
17.72
O
N
H
N
N
N
N
H
200 180 160 140 120 100 80 60 40 20 0 ppm
BRUKER
Current Data Parameters
NAME Hamedan-6
EXPNO 892
PROCNO 1
F2 - Acquisition Parameters
Date_ 20210615
Time 18.54
INSTRUM spect
PROBHD 5 mm Multinucl
PULPROG zgpg
TD 65536
SOLVENT DMSO
NS 199
DS 0
SWH 15060.241 Hz
FIDRES 0.229801 Hz
AQ 2.1758451 sec
RG 1625.5
DW 33.200 usec
DE 6.00 usec
TE 300.0 K
D1 2.00000000 sec
d11 0.03000000 sec
d12 0.00002000 sec
======== CHANNEL f1 ========
NUC1 13C
P1 10.00 usec
PL1 0.00 dB
SFO1 62.9015280 MHz
======== CHANNEL f2 ========
CPDPRG2 waltz16
NUC2 1H
PCPD2 80.00 usec
PL2 3.00 dB
PL12 21.50 dB
PL13 23.00 dB
SFO2 250.1310005 MHz
F2 - Processing parameters
SI 32768
SF 62.8952390 MHz
WDW EM
SSB 0
LB 5.00 Hz
GB 0
PC 1.40

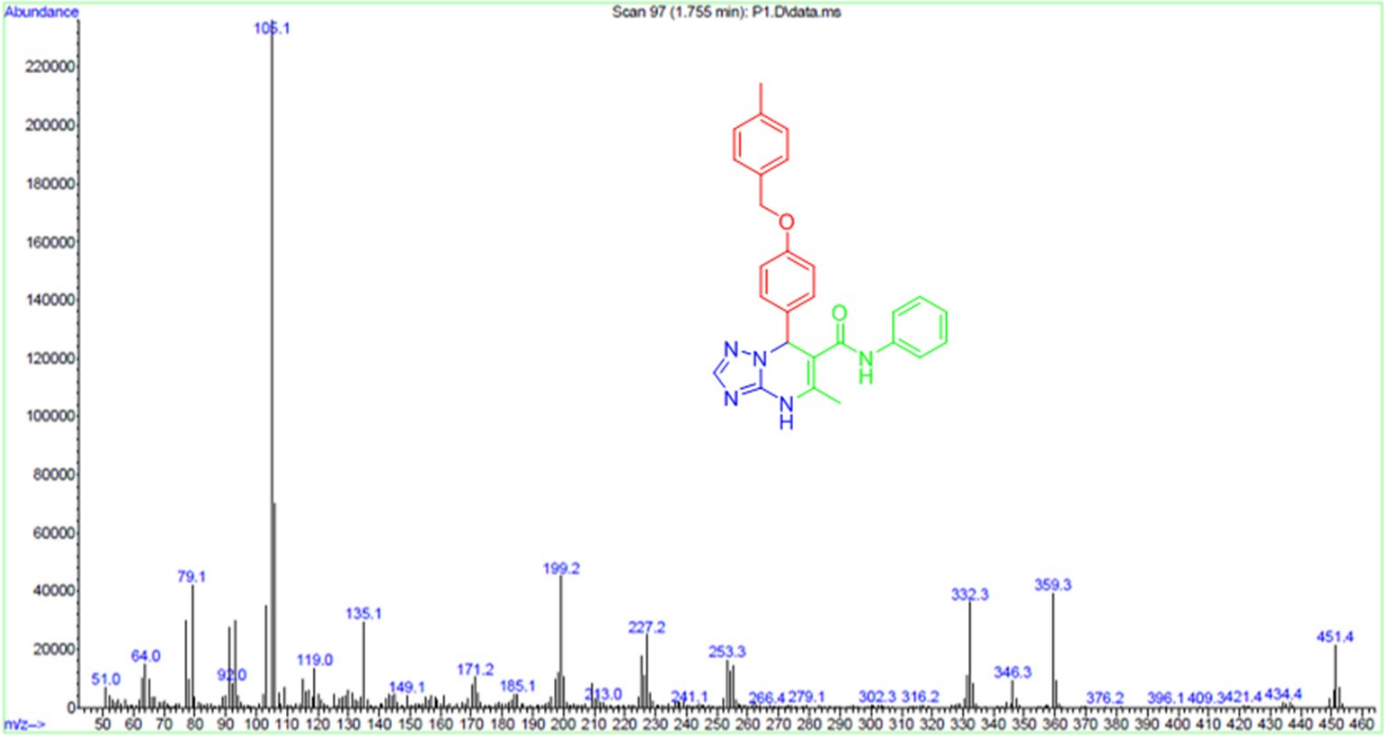

Abundance
Scan 97 (1.755 min): P1.D\data.ms
m/z-->

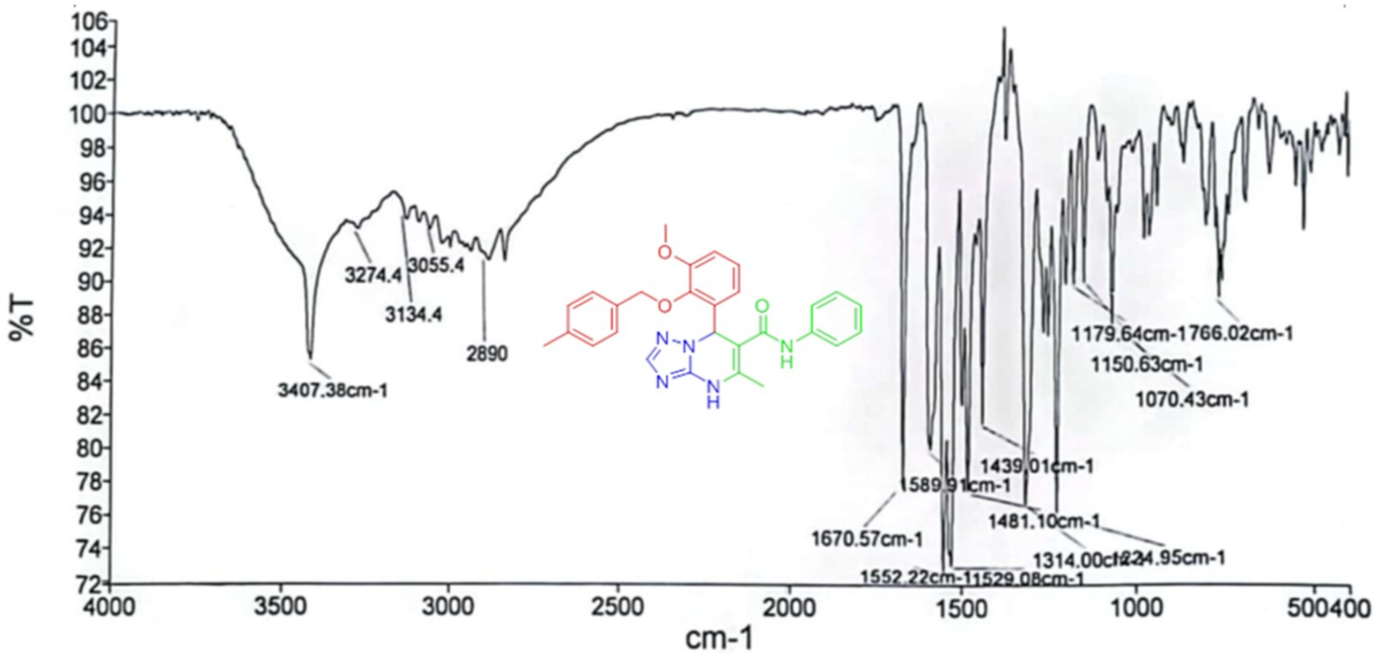
%T
cm-1
3407.38cm-1
3274.4
3134.4
3055.4
2890
1670.57cm-1
1589.91cm-1
1552.22cm-1
1529.08cm-1
1481.10cm-1
1439.01cm-1
1314.00cm-1
1224.95cm-1
1179.64cm-1
766.02cm-1
1150.63cm-1
1070.43cm-1

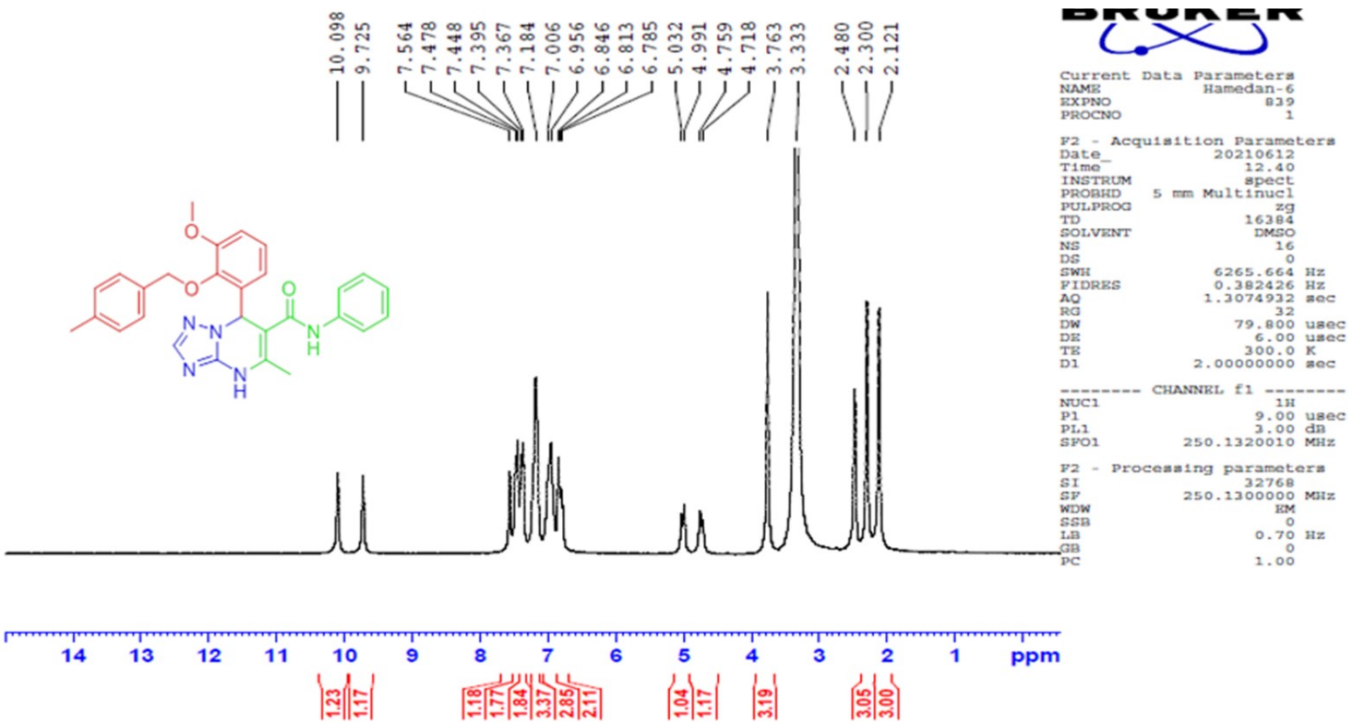
10.098
9.725
7.564
7.478
7.448
7.395
7.367
7.184
7.006
6.956
6.846
6.813
6.785
5.032
4.991
4.759
4.718
3.763
3.333
2.480
2.300
2.121
Current Data Parameters
NAME Hamedan-6
EXPNO 839
PROCNO 1
F2 - Acquisition Parameters
Date_ 20210612
Time 12.40
INSTRUM spect
PROBHD 5 mm Multinucl
PULPROG zg
TD 16384
SOLVENT DMSO
NS 16
DS 0
SWH 6265.664 Hz
FIDRES 0.382426 Hz
AQ 1.3074932 sec
RG 32
DW 79.800 usec
DE 6.00 usec
TE 300.0 K
D1 2.00000000 sec
======== CHANNEL f1 ========
NUC1 1H
P1 9.00 usec
PL1 3.00 dB
SFO1 250.1320010 MHz
F2 - Processing parameters
SI 32768
SF 250.1300000 MHz
WDW EM
SSB 0
LB 0.70 Hz
GB 0
PC 1.00
14 13 12 11 10 9 8 7 6 5 4 3 2 1 ppm
1.23
1.17
1.18
1.77
1.84
3.37
2.85
2.11
1.04
1.17
3.19
3.05
3.00

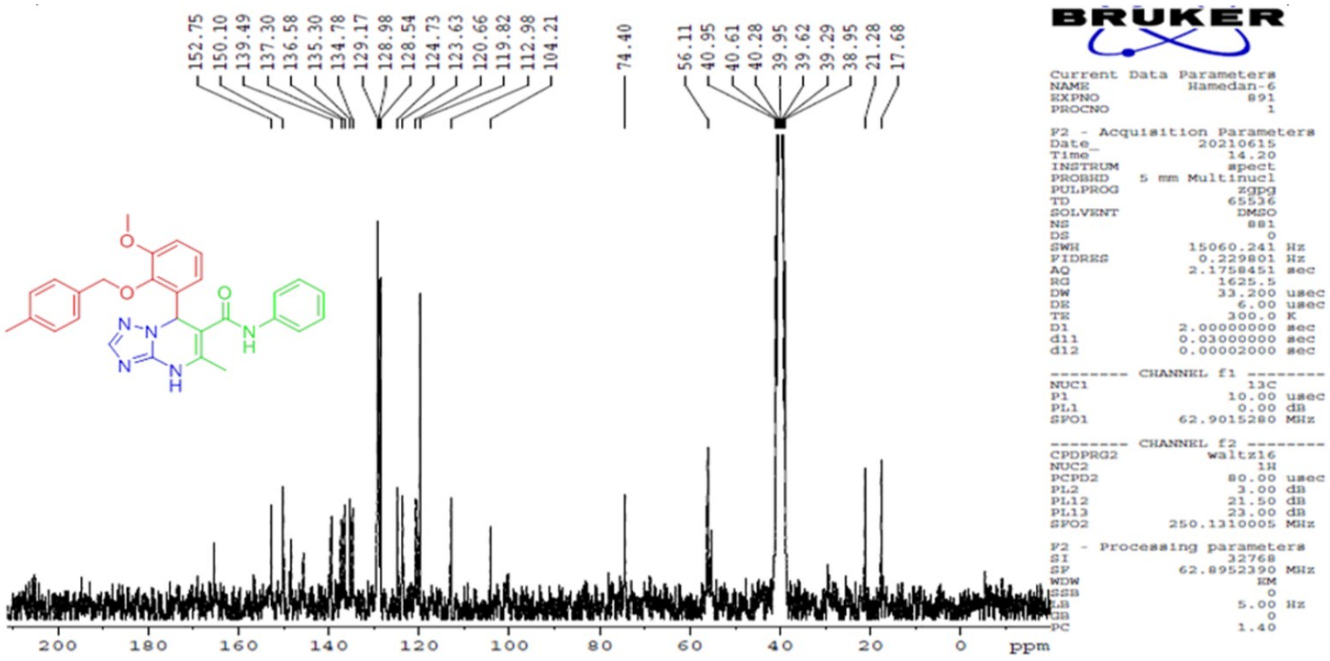
152.75
150.10
139.49
137.30
136.58
135.30
134.78
129.17
128.98
128.54
124.73
123.63
120.66
119.82
112.98
104.21
74.40
56.11
40.95
40.61
40.28
39.95
39.62
39.29
38.95
21.28
17.68
O
O
O
N
N
N
N
H
N
H
BRUKER
Current Data Parameters
NAME Hamedan-6
EXPNO 891
PROCNO 1
F2 - Acquisition Parameters
Date_ 20210615
Time 14.20
INSTRUM spect
PROBHD 5 mm Multinucl
PULPROG zgpg
TD 65536
SOLVENT DMSO
NS 881
DS 0
SWH 15060.241 Hz
FIDRES 0.229801 Hz
AQ 2.1758451 sec
RG 1625.5
DW 33.200 usec
DE 6.00 usec
TE 300.0 K
D1 2.00000000 sec
d11 0.03000000 sec
d12 0.00002000 sec
======== CHANNEL f1 ========
NUC1 13C
P1 10.00 usec
PL1 0.00 dB
SFO1 62.9015280 MHz
======== CHANNEL f2 ========
CPDPRG2 waltz16
NUC2 1H
PCPD2 80.00 usec
PL2 3.00 dB
PL12 21.50 dB
PL13 23.00 dB
SFO2 250.1310005 MHz
F2 - Processing parameters
SI 32768
SF 62.8952390 MHz
WDW EM
SSB 0
LB 5.00 Hz
GB 0
PC 1.40
200 180 160 140 120 100 80 60 40 20 0 ppm

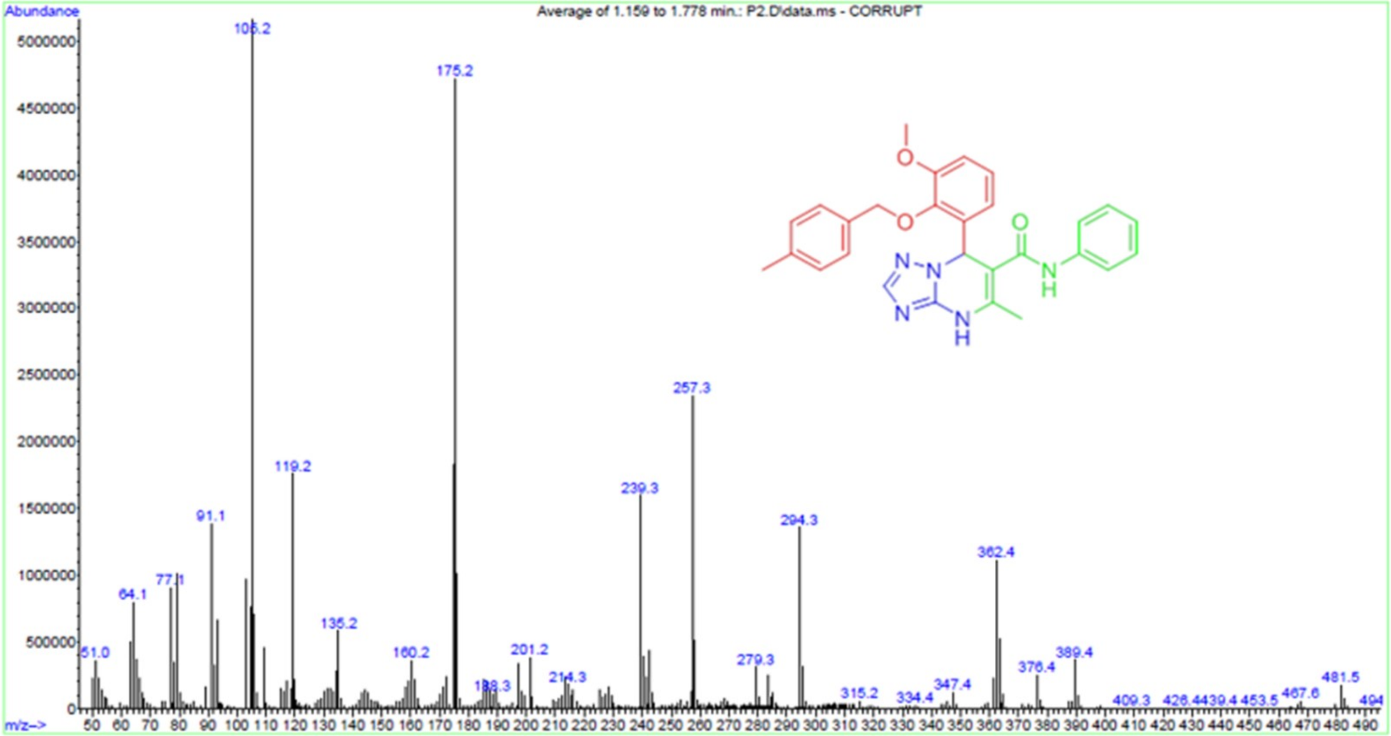

Abundance
Average of 1.159 to 1.778 min.: P2.D\data.ms - CORRUPT
m/z-->

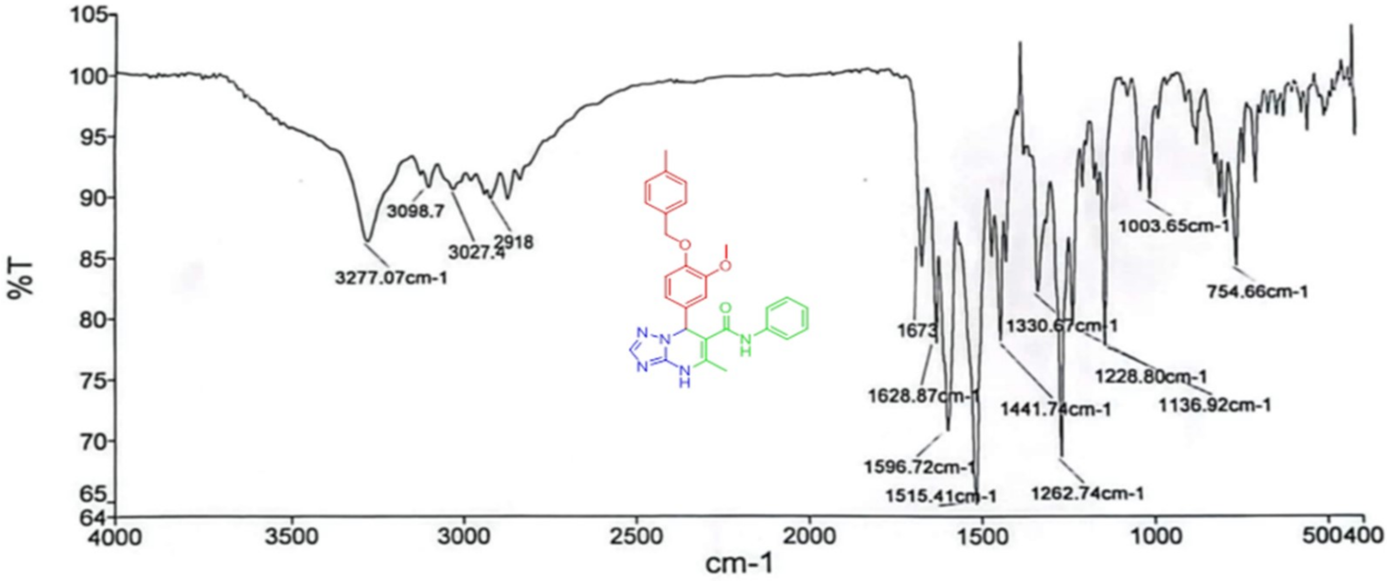
105
100
95
90
85
80
75
70
65
64
%T
3277.07cm-1
3098.7
3027.4
2918
1673
1628.87cm-1
1596.72cm-1
1515.41cm-1
1441.74cm-1
1330.67cm-1
1262.74cm-1
1228.80cm-1
1136.92cm-1
1003.65cm-1
754.66cm-1
4000
3500
3000
2500
2000
1500
1000
500400
cm-1

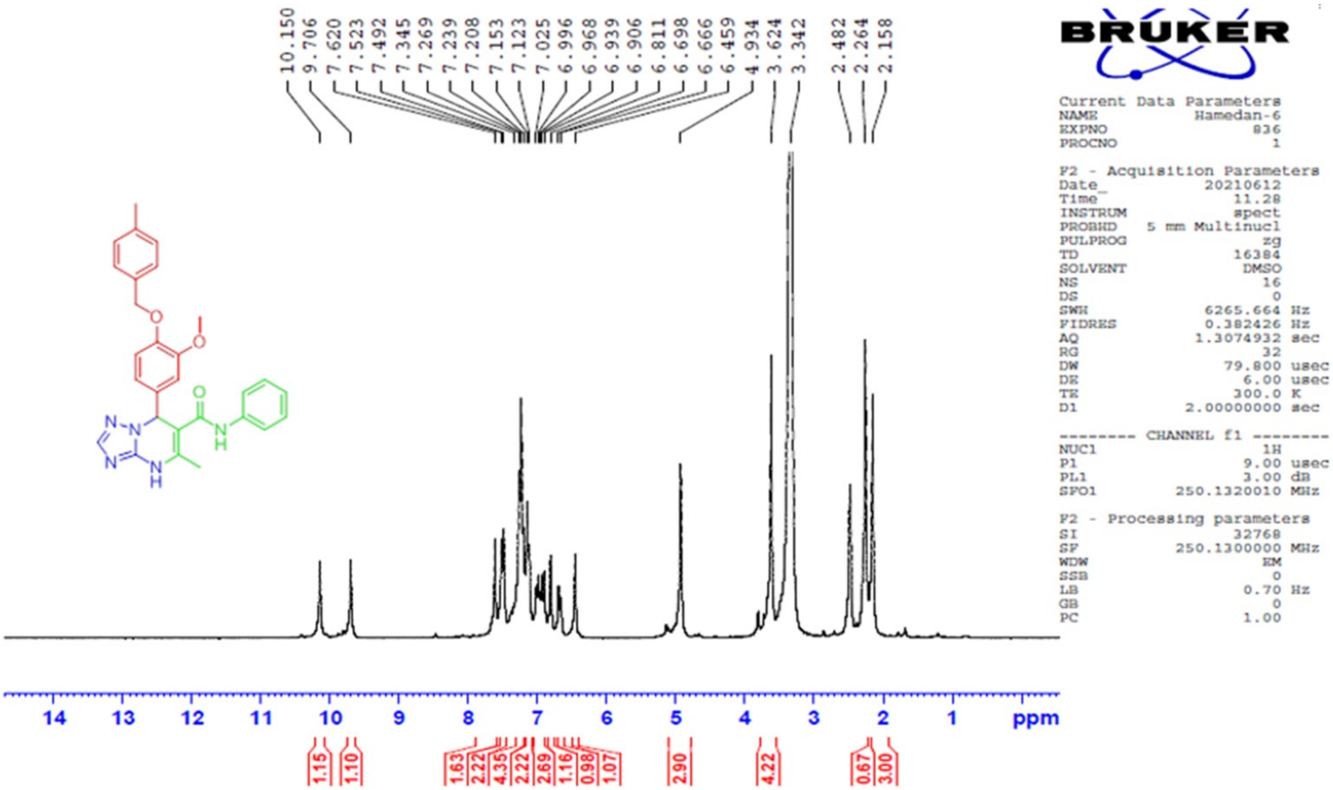
10.150
9.706
7.620
7.523
7.492
7.345
7.269
7.239
7.208
7.153
7.123
7.025
6.996
6.968
6.939
6.906
6.811
6.698
6.666
6.459
4.934
3.624
3.342
2.482
2.264
2.158
BRUKER
Current Data Parameters
NAME Hamedan-6
EXPNO 836
PROCNO 1
F2 - Acquisition Parameters
Date_ 20210612
Time 11.28
INSTRUM spect
PROBHD 5 mm Multinucl
PULPROG zg
TD 16384
SOLVENT DMSO
NS 16
DS 0
SWH 6265.664 Hz
FIDRES 0.382426 Hz
AQ 1.3074932 sec
RG 32
DW 79.800 usec
DE 6.00 usec
TE 300.0 K
D1 2.00000000 sec
======== CHANNEL f1 ========
NUC1 1H
P1 9.00 usec
PL1 3.00 dB
SFO1 250.1320010 MHz
F2 - Processing parameters
SI 32768
SF 250.1300000 MHz
WDW EM
SSB 0
LB 0.70 Hz
GB 0
PC 1.00
14 13 12 11 10 9 8 7 6 5 4 3 2 1 ppm
1.15
1.10
1.63
2.22
4.35
2.22
2.69
1.16
0.98
1.07
2.90
4.22
0.67
3.00

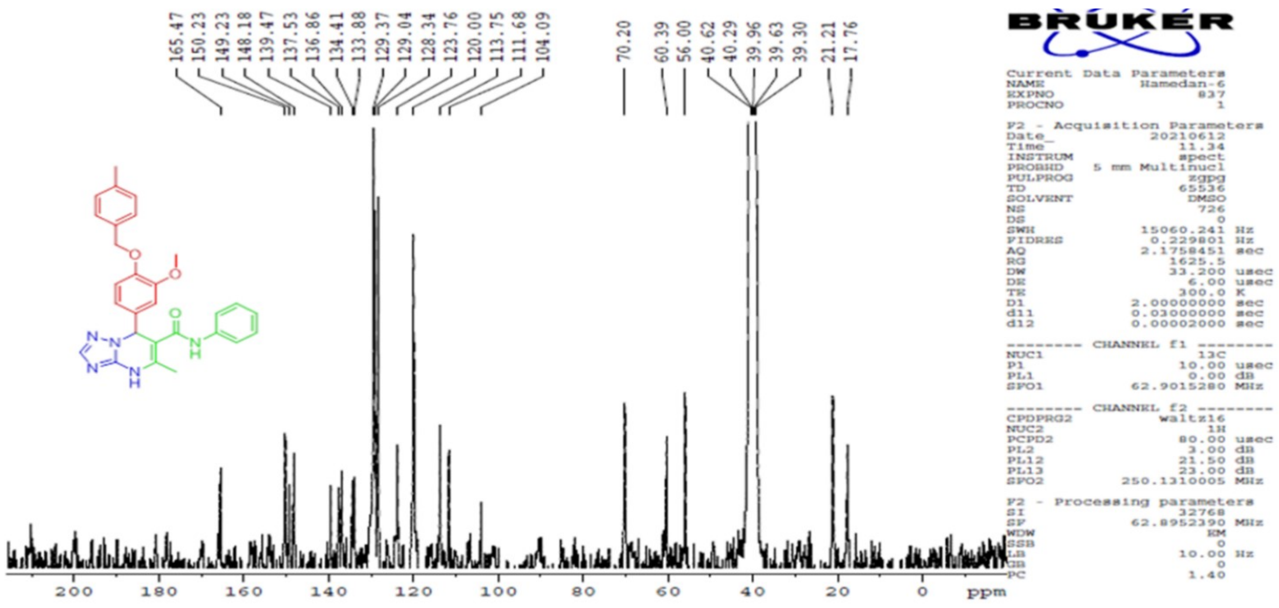
165.47
150.23
149.23
148.18
139.47
137.53
136.86
134.41
133.88
129.37
129.04
128.34
123.76
120.00
113.75
111.68
104.09
70.20
60.39
56.00
40.62
40.29
39.96
39.63
39.30
21.21
17.76
Current Data Parameters
NAME Hamedan-6
EXPNO 837
PROCNO 1
F2 - Acquisition Parameters
Date_ 20210612
Time 11.34
INSTRUM spect
PROBHD 5 mm Multinucl
PULPROG zgpg
TD 65536
SOLVENT DMSO
NS 726
DS 0
SWH 15060.241 Hz
FIDRES 0.229801 Hz
AQ 2.1758451 sec
RG 1625.5
DW 33.200 usec
DE 6.00 usec
TE 300.0 K
D1 2.00000000 sec
d11 0.03000000 sec
d12 0.00002000 sec
======== CHANNEL f1 ========
NUC1 13C
P1 10.00 usec
PL1 0.00 dB
SFO1 62.9015280 MHz
======== CHANNEL f2 ========
CPDPRG2 waltz16
NUC2 1H
PCPD2 80.00 usec
PL2 3.00 dB
PL12 21.50 dB
PL13 23.00 dB
SFO2 250.1310005 MHz
F2 - Processing parameters
SI 32768
SF 62.8952390 MHz
WDW EM
SSB 0
LB 10.00 Hz
GB 0
PC 1.40
200 180 160 140 120 100 80 60 40 20 0 ppm

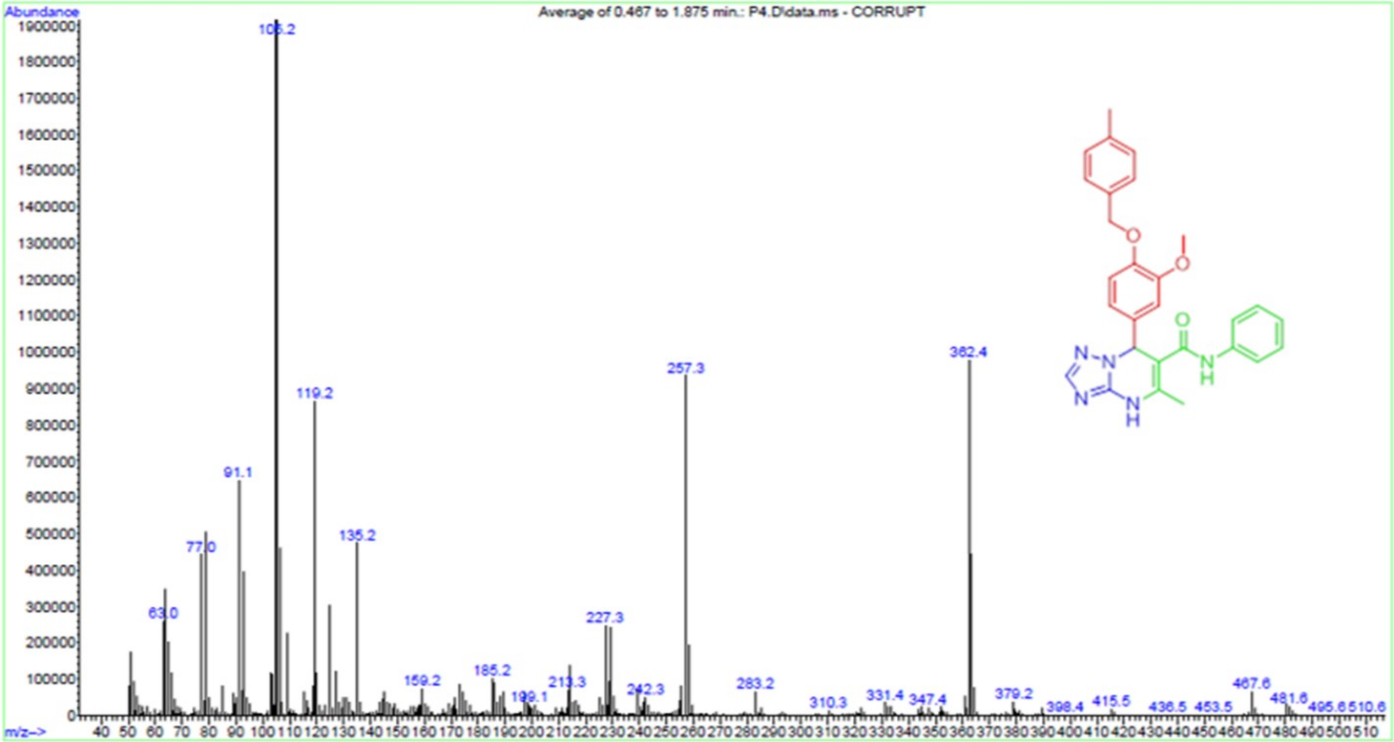
Abundance
Average of 0.467 to 1.875 min.: P4.D\data.ms - CORRUPT
m/z-->
105.2
119.2
91.1
77.0
63.0
135.2
257.3
362.4
227.3
159.2
185.2
199.1
213.3
242.3
283.2
310.3
331.4
347.4
379.2
398.4
415.5
436.5
453.5
467.6
481.6
495.6
510.6

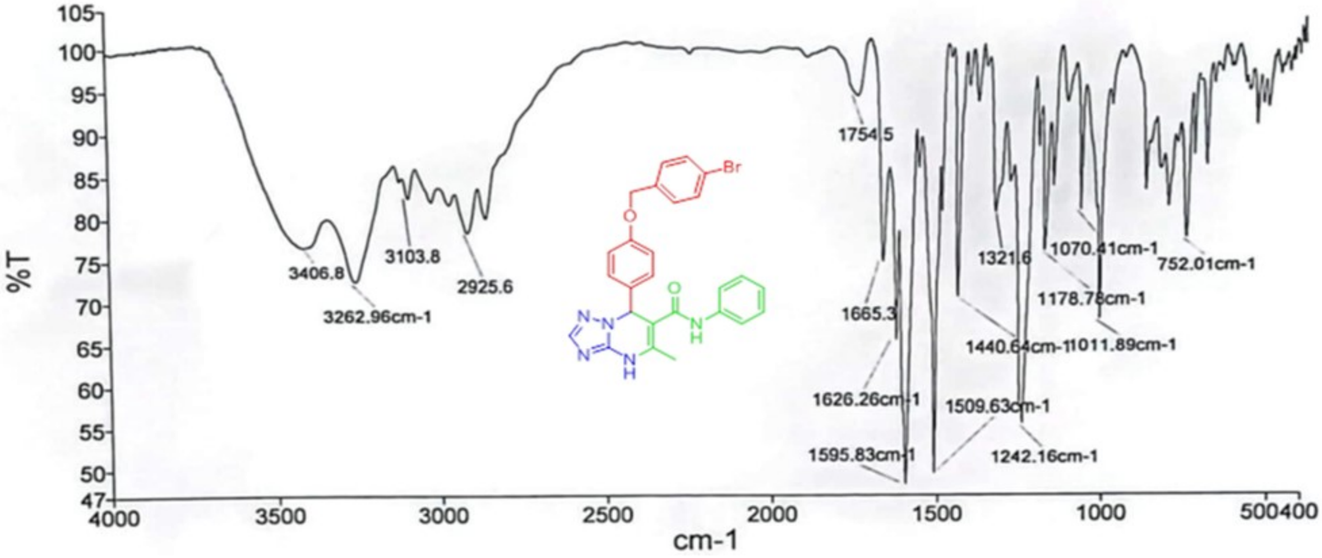
105
100
95
90
85
80
75
70
65
60
55
50
47
%T
4000
3500
3000
2500
2000
1500
1000
500400
cm-1
3406.8
3262.96cm-1
3103.8
2925.6
1754.5
1665.3
1626.26cm-1
1595.83cm-1
1509.63cm-1
1440.64cm-1
1321.6
1242.16cm-1
1178.78cm-1
1070.41cm-1
1011.89cm-1
752.01cm-1
Br
O
N
H

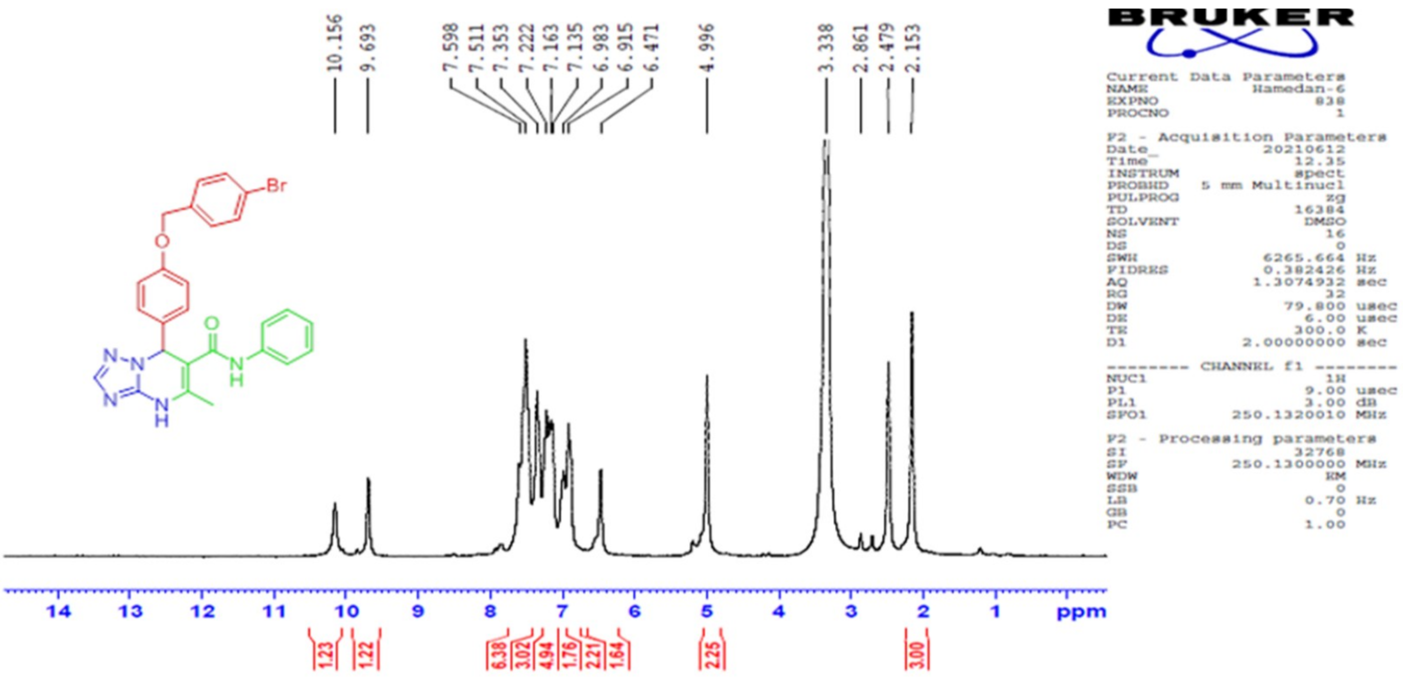
10.156
9.693
7.598
7.511
7.353
7.222
7.163
7.135
6.983
6.915
6.471
4.996
3.338
2.861
2.479
2.153
Br
O
N
N
N
N
H
O
N
H
BRUKER
Current Data Parameters
NAME Hamedan-6
EXPNO 838
PROCNO 1
F2 - Acquisition Parameters
Date_ 20210612
Time 12.35
INSTRUM spect
PROBHD 5 mm Multinucl
PULPROG zg
TD 16384
SOLVENT DMSO
NS 16
DS 0
SWH 6265.664 Hz
FIDRES 0.382426 Hz
AQ 1.3074932 sec
RG 32
DW 79.800 usec
DE 6.00 usec
TE 300.0 K
D1 2.00000000 sec
======== CHANNEL f1 ========
NUC1 1H
P1 9.00 usec
PL1 3.00 dB
SFO1 250.1320010 MHz
F2 - Processing parameters
SI 32768
SF 250.1300000 MHz
WDW EM
SSB 0
LB 0.70 Hz
GB 0
PC 1.00
14 13 12 11 10 9 8 7 6 5 4 3 2 1 ppm
1.23
1.22
6.38
3.02
4.94
1.76
2.21
1.64
2.25
3.00

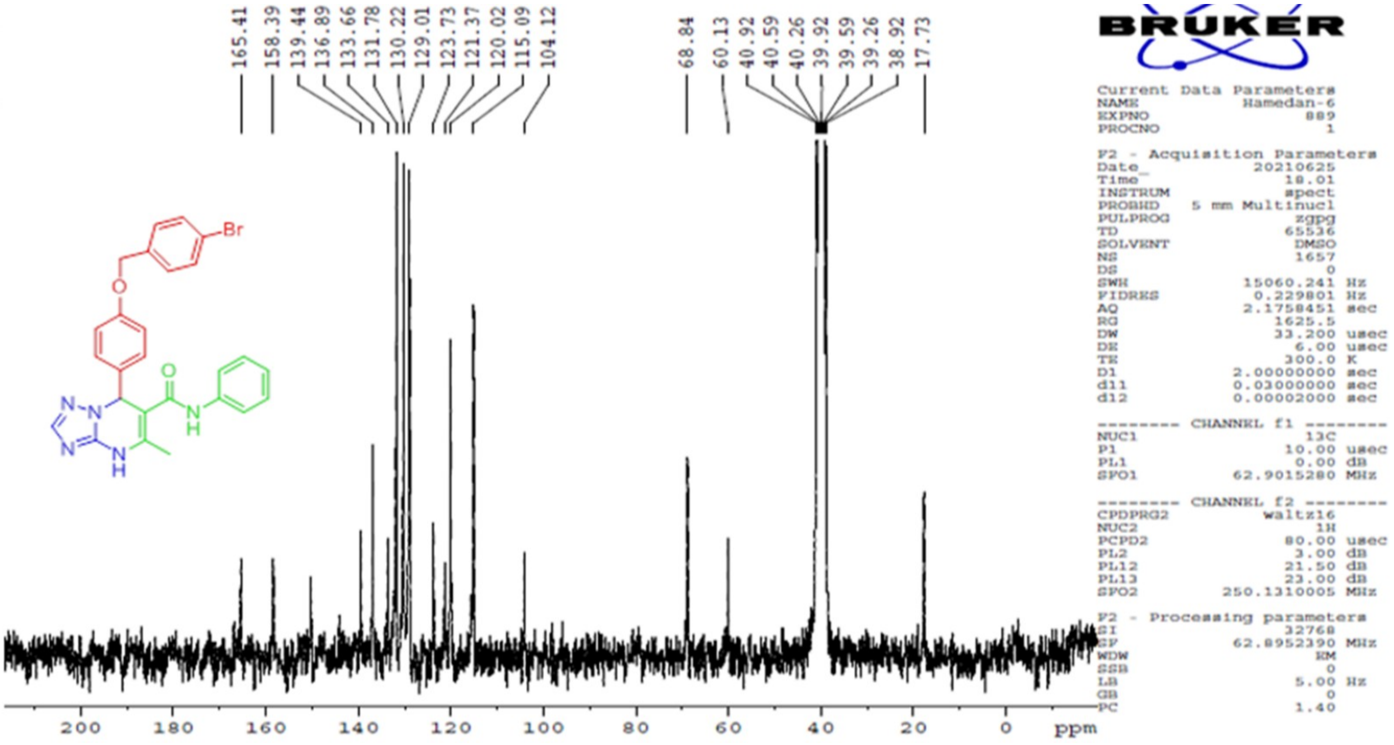

165.41
158.39
139.44
136.89
133.66
131.78
130.22
129.01
123.73
121.37
120.02
115.09
104.12
68.84
60.13
40.92
40.59
40.26
39.92
39.59
39.26
38.92
17.73
Br
O
O
N
N
N
N
H
N
H
BRUKER
Current Data Parameters
NAME Hamedan-6
EXPNO 889
PROCNO 1
F2 - Acquisition Parameters
Date_ 20210625
Time 18.01
INSTRUM spect
PROBHD 5 mm Multinucl
PULPROG zgpg
TD 65536
SOLVENT DMSO
NS 1657
DS 0
SWH 15060.241 Hz
FIDRES 0.229801 Hz
AQ 2.1758451 sec
RG 1625.5
DW 33.200 usec
DE 6.00 usec
TE 300.0 K
D1 2.00000000 sec
d11 0.03000000 sec
d12 0.00002000 sec
======== CHANNEL f1 ========
NUC1 13C
P1 10.00 usec
PL1 0.00 dB
SFO1 62.9015280 MHz
======== CHANNEL f2 ========
CPDPRG2 waltz16
NUC2 1H
PCPD2 80.00 usec
PL2 3.00 dB
PL12 21.50 dB
PL13 23.00 dB
SFO2 250.1310005 MHz
F2 - Processing parameters
SI 32768
SF 62.8952390 MHz
WDW EM
SSB 0
LB 5.00 Hz
GB 0
PC 1.40
200 180 160 140 120 100 80 60 40 20 0 ppm

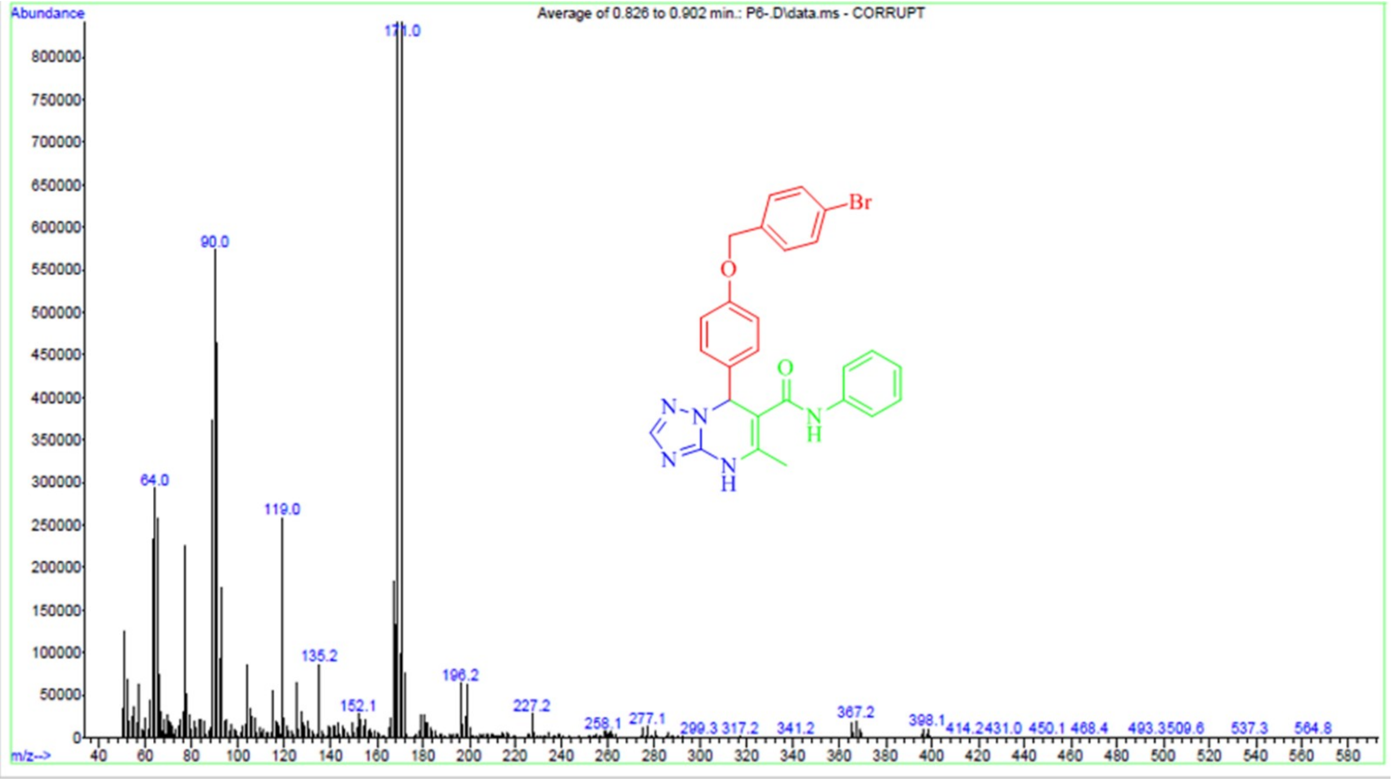

Abundance
Average of 0.826 to 0.902 min.: P6-.D\data.ms - CORRUPT
171.0
90.0
64.0
119.0
135.2
152.1
196.2
227.2
258.1
277.1
299.3
317.2
341.2
367.2
398.1
414.2
431.0
450.1
468.4
493.3
509.6
537.3
564.8
m/z-->
Br
O
N
N
N
N
H
O
N
H

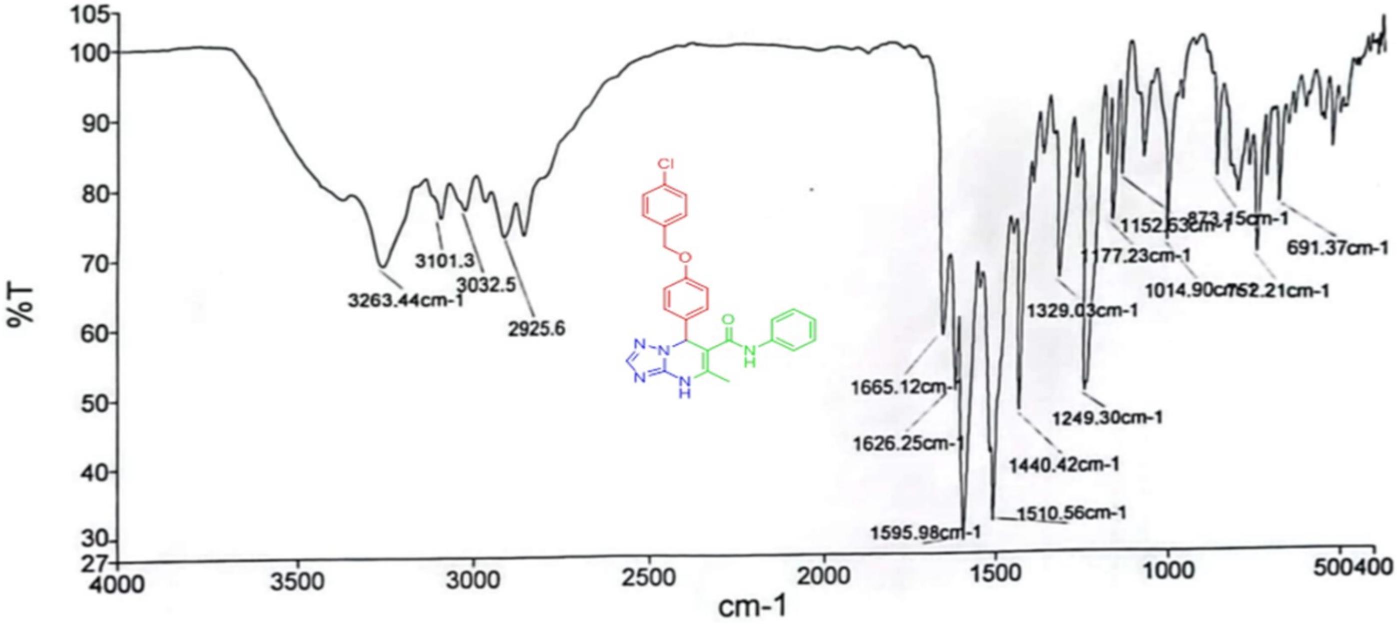

%T
cm-1
3263.44cm-1
3101.3
3032.5
2925.6
1665.12cm-1
1626.25cm-1
1595.98cm-1
1510.56cm-1
1440.42cm-1
1329.03cm-1
1249.30cm-1
1177.23cm-1
1152.63cm-1
1014.90cm-1
873.15cm-1
752.21cm-1
691.37cm-1
Cl
O
N
H

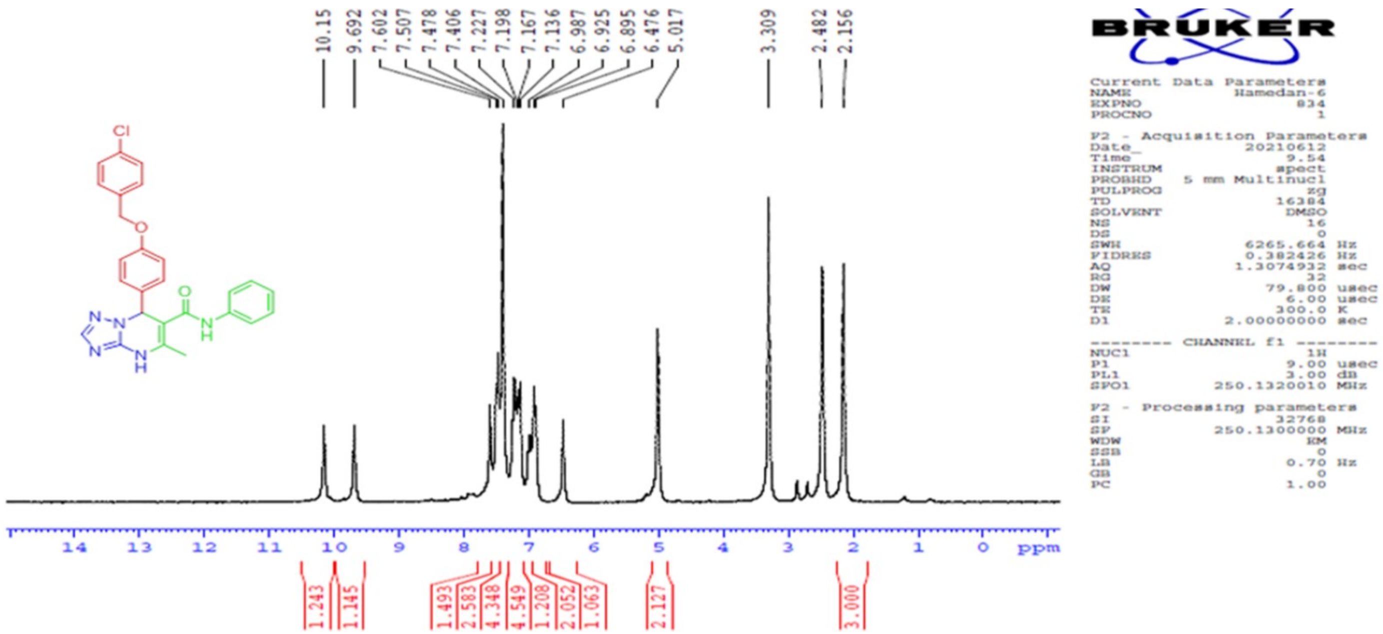
10.15
9.692
7.602
7.507
7.478
7.406
7.227
7.198
7.167
7.136
6.987
6.925
6.895
6.476
5.017
3.309
2.482
2.156
BRUKER
Current Data Parameters
NAME Hamedan-6
EXPNO 834
PROCNO 1
F2 - Acquisition Parameters
Date_ 20210612
Time 9.54
INSTRUM spect
PROBHD 5 mm Multinucl
PULPROG zg
TD 16384
SOLVENT DMSO
NS 16
DS 0
SWH 6265.664 Hz
FIDRES 0.382426 Hz
AQ 1.3074932 sec
RG 32
DW 79.800 usec
DE 6.00 usec
TE 300.0 K
D1 2.00000000 sec
CHANNEL f1
NUC1 1H
P1 9.00 usec
PL1 3.00 dB
SFO1 250.1320010 MHz
F2 - Processing parameters
SI 32768
SF 250.1300000 MHz
WDW EM
SSB 0
LB 0.70 Hz
GB 0
PC 1.00
ppm
1.243
1.145
1.493
2.583
4.348
4.549
1.208
2.052
1.063
2.127
3.000

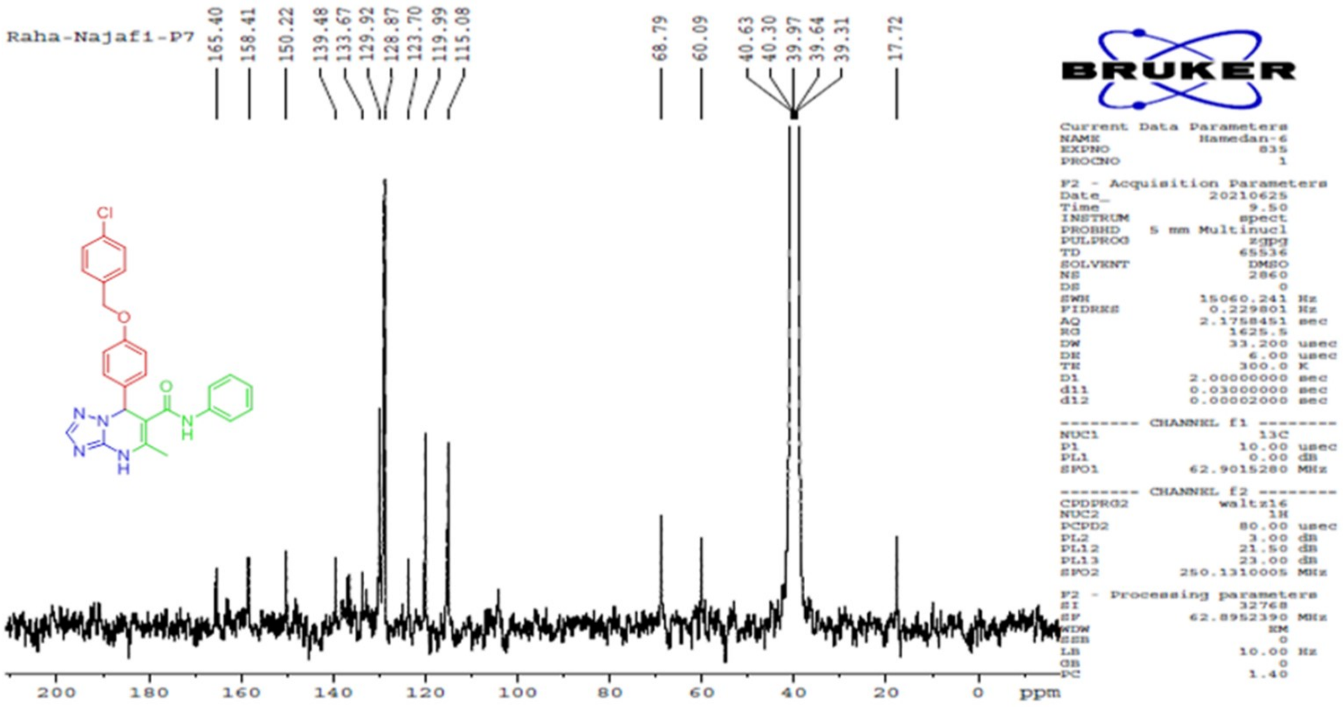
Raha-Najafi-P7
165.40
158.41
150.22
139.48
133.67
129.92
128.87
123.70
119.99
115.08
68.79
60.09
40.63
40.30
39.97
39.64
39.31
17.72
Cl
O
N
N
N
H
O
N
H
BRUKER
Current Data Parameters
NAME Hamedan-6
EXPNO 835
PROCNO 1
F2 - Acquisition Parameters
Date_ 20210625
Time 9.50
INSTRUM spect
PROBHD 5 mm Multinucl
PULPROG zgpg
TD 65536
SOLVENT DMSO
NS 2860
DS 0
SWH 15060.241 Hz
FIDRES 0.229801 Hz
AQ 2.1758451 sec
RG 1625.5
DW 33.200 usec
DE 6.00 usec
TE 300.0 K
D1 2.00000000 sec
d11 0.03000000 sec
d12 0.00002000 sec
======== CHANNEL f1 ========
NUC1 13C
P1 10.00 usec
PL1 0.00 dB
SFO1 62.9015280 MHz
======== CHANNEL f2 ========
CPDPRG2 waltz16
NUC2 1H
PCPD2 80.00 usec
PL2 3.00 dB
PL12 21.50 dB
PL13 23.00 dB
SFO2 250.1310005 MHz
F2 - Processing parameters
SI 32768
SF 62.8952390 MHz
WDW EM
SSB 0
LB 10.00 Hz
GB 0
PC 1.40
200
180
160
140
120
100
80
60
40
20
0
ppm

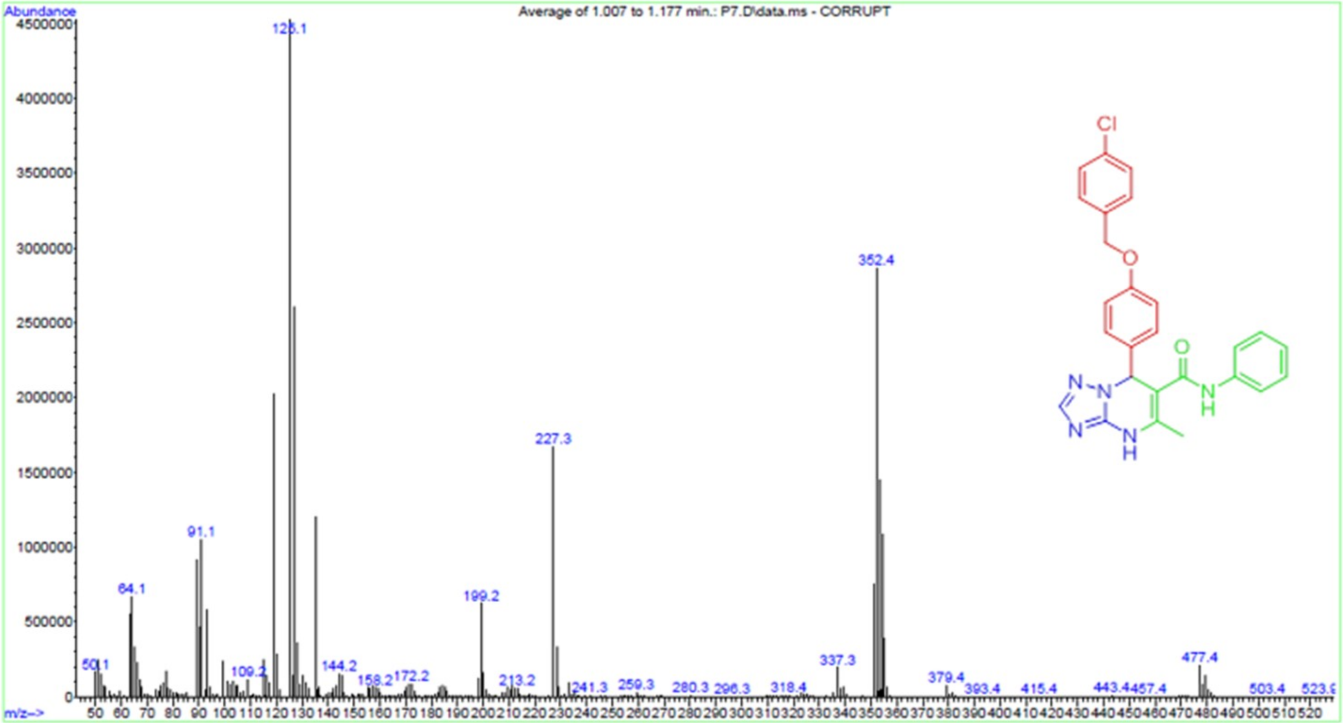
Abundance
Average of 1.007 to 1.177 min.: P7.D\data.ms - CORRUPT
352.4
227.3
91.1
64.1
199.2
337.3
477.4
m/z-->
Cl
O
N
NH
H
N

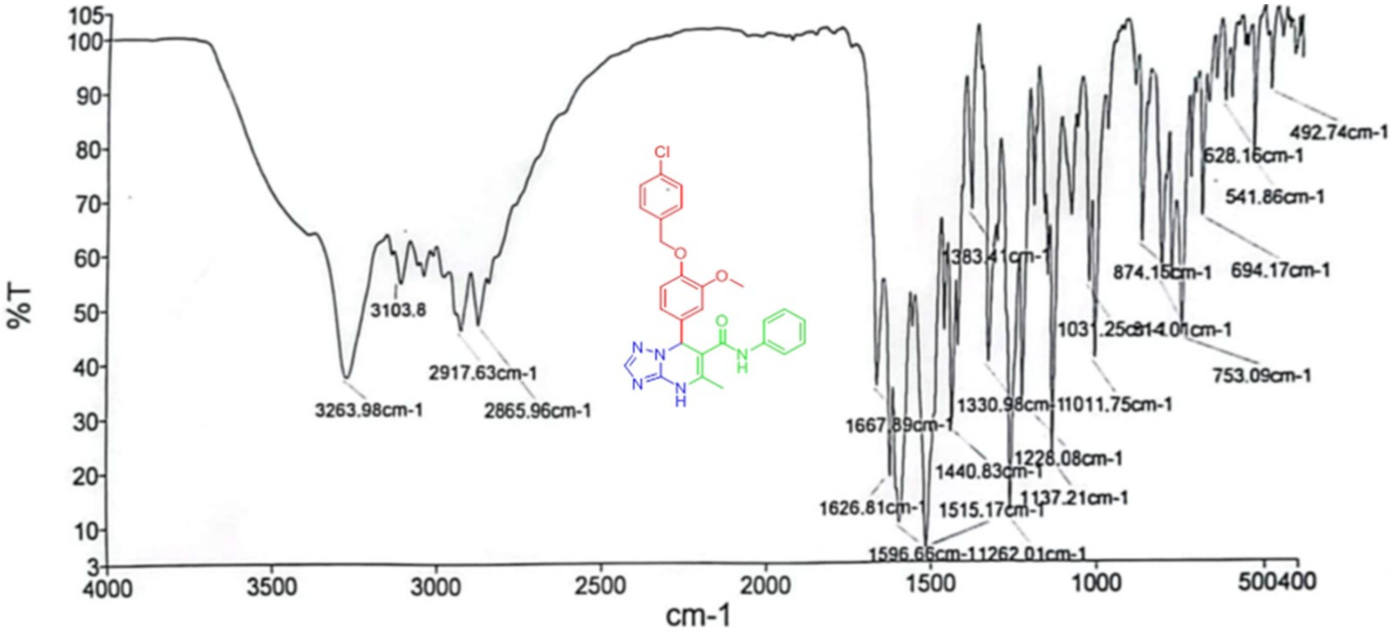
%T
cm-1
3263.98cm-1
3103.8
2917.63cm-1
2865.96cm-1
1667.89cm-1
1626.81cm-1
1596.66cm-11262.01cm-1
1515.17cm-1
1440.83cm-1
1383.41cm-1
1330.98cm-11011.75cm-1
1228.08cm-1
1137.21cm-1
1031.25cm-1
874.15cm-1
814.01cm-1
753.09cm-1
694.17cm-1
628.16cm-1
541.86cm-1
492.74cm-1
Cl
O
N
H

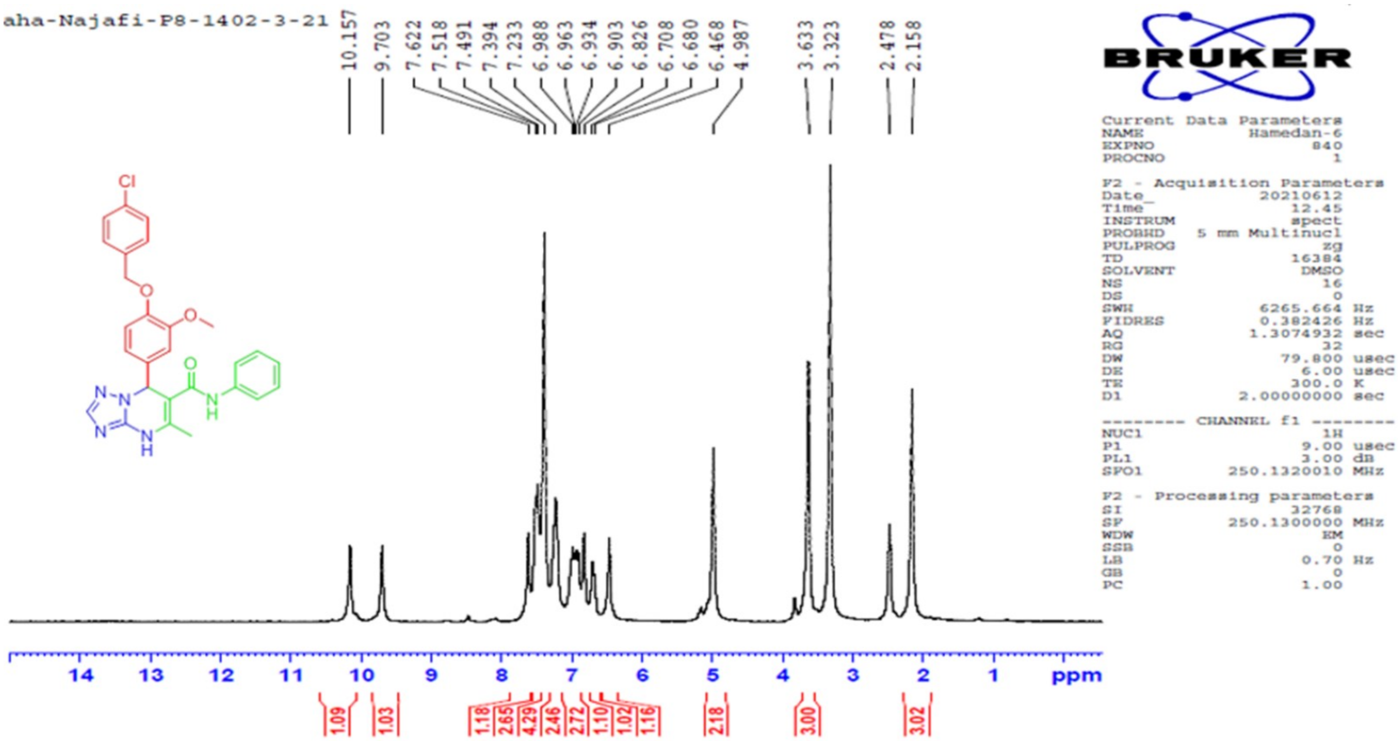
aha-Najafi-P8-1402-3-21
10.157
9.703
7.622
7.518
7.491
7.394
7.233
6.988
6.963
6.934
6.903
6.826
6.708
6.680
6.468
4.987
3.633
3.323
2.478
2.158
BRUKER
Current Data Parameters
NAME Hamedan-6
EXPNO 840
PROCNO 1
F2 - Acquisition Parameters
Date_ 20210612
Time 12.45
INSTRUM spect
PROBHD 5 mm Multinucl
PULPROG zg
TD 16384
SOLVENT DMSO
NS 16
DS 0
SWH 6265.664 Hz
FIDRES 0.382426 Hz
AQ 1.3074932 sec
RG 32
DW 79.800 usec
DE 6.00 usec
TE 300.0 K
D1 2.00000000 sec
======== CHANNEL f1 ========
NUC1 1H
P1 9.00 usec
PL1 3.00 dB
SFO1 250.1320010 MHz
F2 - Processing parameters
SI 32768
SF 250.1300000 MHz
WDW EM
SSB 0
LB 0.70 Hz
GB 0
PC 1.00
Cl
O
N
NH
14
13
12
11
10
9
8
7
6
5
4
3
2
1
ppm
1.09
1.03
1.18
2.65
4.29
2.46
2.72
1.10
1.02
1.16
2.18
3.00
3.02

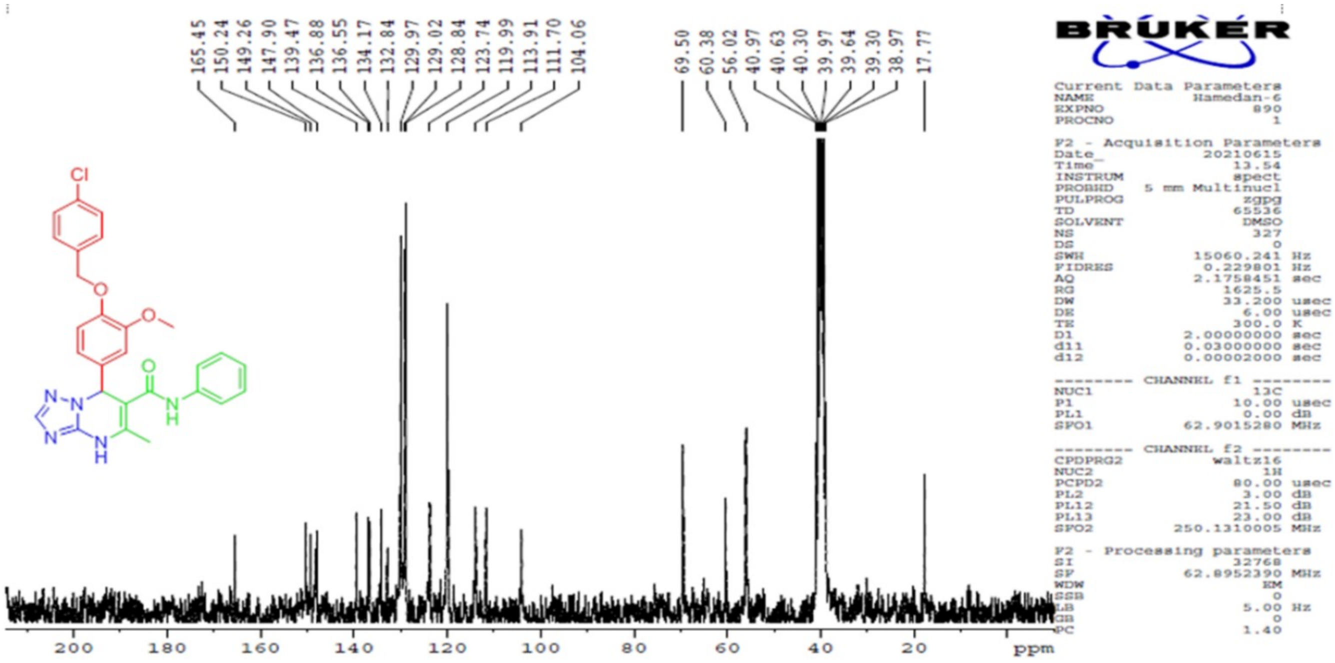
165.45
150.24
149.26
147.90
139.47
136.88
136.55
134.17
132.84
129.97
129.02
128.84
123.74
119.99
113.91
111.70
104.06
69.50
60.38
56.02
40.97
40.63
40.30
39.97
39.64
39.30
38.97
17.77
BRUKER
Current Data Parameters
NAME Hamedan-6
EXPNO 890
PROCNO 1
F2 - Acquisition Parameters
Date_ 20210615
Time 13.54
INSTRUM spect
PROBHD 5 mm Multinucl
PULPROG zgpg
TD 65536
SOLVENT DMSO
NS 327
DS 0
SWH 15060.241 Hz
FIDRES 0.229801 Hz
AQ 2.1758451 sec
RG 1625.5
DW 33.200 usec
DE 6.00 usec
TE 300.0 K
D1 2.00000000 sec
d11 0.03000000 sec
d12 0.00002000 sec
======== CHANNEL f1 ========
NUC1 13C
P1 10.00 usec
PL1 0.00 dB
SFO1 62.9015280 MHz
======== CHANNEL f2 ========
CPDPRG2 waltz16
NUC2 1H
PCPD2 80.00 usec
PL2 3.00 dB
PL12 21.50 dB
PL13 23.00 dB
SFO2 250.1310005 MHz
F2 - Processing parameters
SI 32768
SF 62.8952390 MHz
WDW EM
SSB 0
LB 5.00 Hz
GB 0
PC 1.40
200 180 160 140 120 100 80 60 40 20 ppm

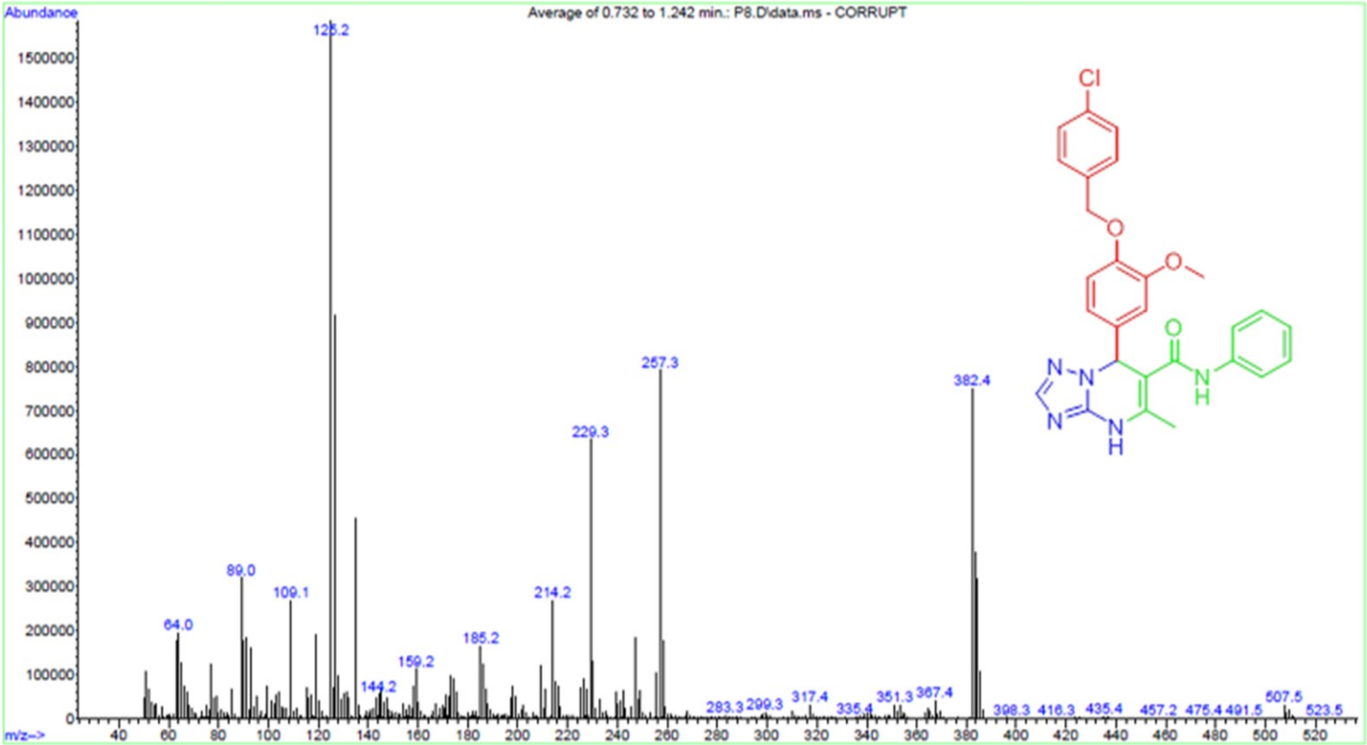
Abundance
Average of 0.732 to 1.242 min.: P8.D\data.ms - CORRUPT
m/z-->
Cl
O
O
N
N
N
N
H
O
N
H
